# Relation of Statin Use with Esophageal Cancer

**DOI:** 10.3390/ph16060900

**Published:** 2023-06-19

**Authors:** Sarang Jang, Hyo Geun Choi, Mi Jung Kwon, Ji Hee Kim, Joo-Hee Kim, So Young Kim

**Affiliations:** 1 Department of Public Health, Sahmyook University, Seoul 01795, Republic of Korea; jangsarang@gmail.com; 2 Mdanalytics, Seoul 06349, Republic of Korea; pupen@naver.com; 3 Suseoseoulent Clinic, Seoul 06349, Republic of Korea; 4 Department of Pathology, Hallym University Sacred Heart Hospital, Hallym University College of Medicine, Anyang 14068, Republic of Korea; mulank99@hallym.or.kr; 5 Department of Neurosurgery, Hallym University Sacred Heart Hospital, Hallym University College of Medicine, Anyang 14068, Republic of Korea; kimjihee@hallym.or.kr; 6 Department of Medicine, Hallym University Sacred Heart Hospital, Hallym University College of Medicine, Anyang 14068, Republic of Korea; luxjhee@hallym.or.kr; 7 Department of Otorhinolaryngology-Head & Neck Surgery, CHA Bundang Medical Center, CHA University, Seongnam 13496, Republic of Korea

**Keywords:** statins, esophageal cancer, risk factors, case-control studies, epidemiology

## Abstract

The present study evaluated the association of long-term statin use with the diagnosis and mortality of esophageal cancer in a Korean population. The Korean National Health Insurance Service-Health Screening Cohort from 2002 to 2019 was enrolled. Esophageal cancer patients were matched with control participants for demographic variables. The statin prescription histories were collected and grouped into <180 days, 180 to 545 days, and >545 days of duration. Propensity score overlap weighting was applied to minimize the bias between the esophageal cancer and control groups. The odds ratios (ORs) of the duration of statin use for esophageal cancer were analyzed using propensity score overlap weighted multivariable logistic regression analysis. The esophageal cancer group was classified as dead and surviving patients, and the ORs of the duration of statin use for the mortality of esophageal cancer were analyzed in an identical manner. Secondary analyses were conducted according to comorbid factors. Patients with esophageal cancer did not show lower odds for the duration of statin prescription in the overall study population (OR = 1.30, 95% CI = 1.03–1.65, *p* = 0.027 for 180 to 545 days and OR = 1.29, 95% CI = 1.08–1.55, *p* = 0.006 for >545 days). Subgroups of nonsmokers, past and current smokers, alcohol consumption ≥ 1 time a week, SBP < 140 mmHg and DBP < 90 mmHg, fasting blood glucose ≥ 100 mg/dL, total cholesterol ≥ 200 mg/dL, CCI score = 0, and nondyslipidemia history demonstrated low odds for the duration of statin prescription. Both types of statins, hydrophilic and lipophilic statins, were not related to a lower rate of esophageal cancer. The mortality of esophageal cancer was not associated with the duration of statin prescription. A subgroup with total cholesterol ≥ 200 mg/dL showed lower odds of statin prescription for mortality of esophageal cancer. The duration of statin prescription was not related to a lower rate or mortality of esophageal cancer in the adult Korean population.

## 1. Introduction

Esophageal cancer is one of the main causes of mortality, with an estimated 5.48 mortality cases per 100,000 persons in the general population [1]. Men and old age groups were the most susceptible populations for esophageal cancer [1]. Although the prevalence of esophageal cancer has been decreasing in Korea, the detection rate of early-stage esophageal cancer has been rising in recent years [2]. Therefore, prevention and therapeutics for esophageal cancer have been the focus of many researchers. In addition to surgery in the early stage of esophageal cancer, chemoradiation and immunotherapy have been used to improve the survival outcome for esophageal cancer [3,4]. However, mortality from esophageal cancer is still considerable, ranking as the sixth highest cause of death from cancer [3]. There are several risk factors for esophageal cancer [5]. Low socioeconomic status, smoking, alcohol consumption, and micronutrient deficiencies have been acknowledged as risk factors for the squamous cell type of esophageal cancer [6]. Barrett’s esophagus, gastroesophageal reflux disease, obesity, and smoking have been reported to increase the risk of adenocarcinoma cell-type esophageal cancer [6].

Statins are lipid-lowering drugs that inhibit 3-hydroxy-3-methylglutaryl coenzyme A reductase, thereby interfering with cholesterol synthesis [7]. In addition to these effects, research has acknowledged the pleiotropic effects of statins not only in the cardiovascular system but also in cancer [7]. A number of in vitro experiments have proposed that statins inhibit proliferation and lead to apoptosis in esophageal cancer cells [8,9]. In addition, several epidemiologic studies have reported reduced mortality from esophageal cancer when patients take statins after the diagnosis of esophageal cancer [10,11,12]. A meta-analysis estimated that statin medication reduced mortality by approximately 16% in esophageal cancer patients [12].

Although many previous studies have implied the protective or adjunctive effects of statins in esophageal cancer patients, to our knowledge, the impacts of long-term statin use in esophageal cancer have not been investigated. It can be supposed that statins may have additive preventive effects on the development of esophageal cancer. We hypothesized that statins could have preventive effects on esophageal cancer based on previous pre-clinical and clinical studies. Because treatment of esophageal cancer is still challenging and esophageal cancer accounts for a high rate of mortality, it will be clinically very valuable if statins have preventive effects on esophageal cancer. Moreover, prior research has rarely considered both the incidence and prognosis or mortality of esophageal cancer for statin medication. In this study, statin use before the diagnosis of esophageal cancer was analyzed. By analyzing both the incidence and mortality of esophageal cancer, we can estimate the preventive and adjustive impacts of statins in patients with esophageal cancer.

## 2. Results

The patients with esophageal cancer showed differences in obesity, smoking status, alcohol consumption, SBP, DBP, total cholesterol level, fasting blood glucose level, CCI score, and dyslipidemia compared with control participants (Table 1, standardized difference [sd] > 0.01). Because these variables have been reported to be associated with morbidities requiring statin medication, an overlap weighting adjustment was conducted. After the overlap weighting adjustment, all variables included in the analysis, age, sex, income, region of residence, obesity, smoking status, alcohol consumption, SBP, DBP, total cholesterol level, fasting blood glucose level, CCI score, and dyslipidemia, demonstrated standardized differences of 0.00 between the esophageal cancer group and control group.

In the esophageal cancer group, 82.9% (914/1102), 5.9% (65/1102), and 11.2% (123/1102) of esophageal cancer patients demonstrated histories of <180 days, 180 to 545 days, and >545 days of statin prescription, respectively (Table 2). Statin medication was not related to the risk of esophageal cancer in the crude analysis (all *p* > 0.05). However, in the overlap-weighted model, a longer duration of statin medications was related to a higher risk of esophageal cancer (OR = 1.30, 95% CI = 1.03–1.65, *p* = 0.027 for 180 to 545 days and OR = 1.29, 95% CI = 1.08–1.55, *p* = 0.006 for >545 days).

In subgroup analyses, <65 years old, ≥65 years old, men, low income, urban and rural residents, normal weight, overweight, and CCI score = 1 groups indicated higher odds for esophageal cancer according to the long duration of statin prescription (Table S1 and Figure S1). On the other hand, subgroups of nonsmokers, past and current smokers, alcohol consumption ≥ 1 time a week, SBP < 140 mmHg and DBP < 90 mmHg, fasting blood glucose ≥ 100 mg/dL, total cholesterol ≥ 200 mg/dL, CCI score = 0, and nondyslipidemia history demonstrated low odds of esophageal cancer related to a long duration of statin prescription.

According to the types of statins, both hydrophilic and lipophilic statin prescriptions were related to the risk of esophageal cancer in the overlap-weighted model (OR = 1.45, 95% CI = 1.08–1.94, *p* = 0.013 for hydrophilic statin prescription for 180 to 545 days and OR = 1.40, 95% CI = 1.08–1.82, *p* = 0.012 for lipophilic statin prescription for >545 days). In subgroup analyses, the relationship of each type of statin with esophageal cancer was heterogeneous according to the subgroups. Both hydrophilic and lipophilic statins demonstrated high or low odds for esophageal cancer in many subgroups (Tables S2 and S3, Figures S2 and S3).

In the esophageal cancer group, the patients who died were compared with patients who survived statin prescriptions. Age, sex, income, region of residence, obesity, smoking status, alcohol consumption, SBP, DBP, total cholesterol, fasting blood glucose, CCI score, dyslipidemia history, and types of treatment were different between the non-surviving and surviving groups (Table 3). After overlap weighting adjustment, all these variables were equalized between the deceased and survived groups (all sd = 0.00).

The duration of statin prescription was not related to the mortality of esophageal cancer (Table 4, all *p* > 0.05). Neither hydrophilic nor lipophilic statins were associated with the mortality of esophageal cancer (all *p* > 0.05).

Among the subgroups, the total cholesterol ≥ 200 mg/dL group showed low odds for mortality of esophageal cancer in patients with 180 to 545 days of statin prescription (OR = 0.36, 95% CI = 0.14–0.95, *p* = 0.038, Table S4 and Figure S4). In addition, the high-income group demonstrated low odds for mortality of esophageal cancer in patients with >545 days of hydrophilic statin prescription (OR = 0.32, 95% CI = 0.12–0.85, *p* = 0.023, Table S5 and Figure S5). On the other hand, lipophilic statin prescription was associated with high odds for the mortality of esophageal cancer in the <65 years old, low-income, SBP < 140 mmHg and DBP < 90 mmHg, and total cholesterol < 200 mg/dL groups (Table S6 and Figure S6).

## 3. Discussion

A long duration of statin use was related to increased odds of esophageal cancer in the adult Korean population. Both hydrophilic and lipophilic statins demonstrated high odds of esophageal cancer related to a long duration of statin use. The mortality of esophageal cancer was not related to the long duration of statin use in the overall adult Korean population. However, in subgroups, the mortality of esophageal cancer was lower in long-duration hydrophilic statin users, while it was high in long-duration lipophilic statin users. In summary, long-term statin use was not associated with the diagnosis and mortality of esophageal cancer, although hydrophilic statin use was related to the low mortality of esophageal cancer in a specific subgroup. Although there have been numerous studies suggesting the therapeutic effects of statins on esophageal cancer, the current study improved the previous knowledge of different associations of statin use with esophageal cancer in different ethnic populations.

Long-term statin medication was not associated with a lower risk of esophageal cancer in the present study. On the other hand, several previous studies have suggested the protective effects of statins on esophageal cancer [13,14]. In addition to esophageal cancer, the anti-cancer effect of statins has been supposed in other types of cancers, such as hepatocellular carcinoma [15]. Statins were suggested to have anti-oncogenic effects by impeding the expression of proto-oncogenes and promoting apoptotic pathways [15]. In esophageal cancer, a meta-analysis estimated that statin use was associated with a 43% lower rate of development of esophageal adenocarcinoma in patients with Barrett’s esophagus (95% CI = 0.43–0.75) [13]. Moreover, another meta-analysis predicted an 18% lower incidence of all esophageal cancers related to statin use (95% CI = 0.7–0.88) [14]. However, the types, doses, and duration of statin use were not specified in previous studies, although it was expected that there might be a dose- or duration-dependent inverse association between statin use and the incidence of esophageal cancer [14]. Thus, long-term statin use can have a different relationship with the incidence of esophageal cancer, as shown in the present study. In this study, the incidence of esophageal cancer was higher in statin users. This result may be dependent on the high rate of comorbidities in esophageal cancer patients, which results in a high rate of statin prescriptions. It is also possible that underlying esophageal diseases, such as Barrett’s esophagus or esophageal ulcers, which need statin medication, can be more prevalent in patients with esophageal cancer. Because there were some subgroups that showed a lower incidence of esophageal cancer related to statin use, we cannot conclude that statin use can increase the risk of esophageal cancer in this study.

Long-term statin medication did not decrease the mortality of esophageal cancer in the overall adult population in this study. Many prior studies supposed the reduced mortality of esophageal cancer following statin medication [10,11,12,16,17,18,19]. A nationwide population study in the UK demonstrated a longer median survival time in esophageal cancer patients who used statins after the diagnosis of esophageal cancer (14.9 months [interquartile range, IQR = 7.1–52.3 months] in the statin use group vs. 8.1 months [IQR = 3.3–23.2 months] in the no statin use group) [10]. However, according to the types of esophageal cancer, esophageal squamous cell carcinoma did not show reduced mortality associated with statin use. Thus, it can be presumed that the protective effect of statins can be valid in specific types of esophageal cancer. Another retrospective study also demonstrated the low mortality of esophageal cancer (hazard ratio = 0.79, 95% CI = 0.70–0.88) [11]. However, statin medication before esophageal cancer did not reduce the diagnosis of esophageal cancer. Thus, the authors speculated that advanced-stage or highly morbid esophageal cancer patients might not be prescribed statins, which may result in a lower rate of statin medication in mortality cases [11].

The partial or minimal protective effects of statins on esophageal cancer in the present study can originate from the differential effects of statins on esophageal cancer according to the histologic types of esophageal cancer. A previous study demonstrated that the protective effect of statins on esophageal cancer was limited to the adenocarcinoma type of esophageal cancer but not the squamous type of esophageal cancer. In the Asian population, squamous esophageal cancer is the predominant histologic type of esophageal cancer [2,20]. Thus, the protective effect of statins in the Korean adult population may be limited.

This is based on a nationwide, large population database, which strengthens the statistical power of this study. We selected control participants who were matched for demographic factors, and overlap weighting adjustment was conducted to minimize potential bias due to confounders. In addition, secondary analyses were extensively performed to specify the association of statin use with the diagnosis or mortality of esophageal cancer in specific subgroups. However, certain limitations need to be considered when interpreting the present results. The esophageal cancer group in this study included a wide spectrum of cancer patients in terms of histologic types, stages, and treatment modalities. For statin medication, we did not count the dose of statin medications in the study population. Because this study used a health claim database, statin use was collected based on the prescription of medication. However, patients’ compliance with statin prescriptions can vary among participants. Although numerous variables were included in the analyses, potential unconsidered confounders cannot be excluded in the present study. For instance, psychological factors, such as stress, depression, and anxiety, and dietary factors that can impact the metabolism of statins can impact both statin use and esophageal cancer. Last, this study exclusively enrolled the Korean population. Therefore, ethnic and regional differences can exist in the relationship between statin use and esophageal cancer.

## 4. Methods

### 4.1. Ethics

The ethics committee of Hallym University (2019-10-023) permitted the current investigation. Written informed consent was exempted by the Institutional Review Board.

### 4.2. Study Population and Participant Selection

The Korean National Health Insurance Service-Health Screening Cohort data from 2002 through 2019 were used.

Esophageal cancer participants were selected from 514,866 participants with 895,300,177 medical claim codes from 2002 through 2019 (*n* = 1149). The control group was selected from the participants who were not diagnosed with esophageal cancer during the identical study period (*n* = 513,717). The participants who were diagnosed with malignant neoplasms of digestive organs (ICD-10 codes: C15-C26) ≥ 2 times (*n* = 39,414), and if they were diagnosed with esophageal cancer (ICD-10 codes: C15) < 3 times (*n* = 433) were removed. Esophageal cancer participants and control participants were 1:4 matched. The control participants were selected in random order.

**Figure 1:**
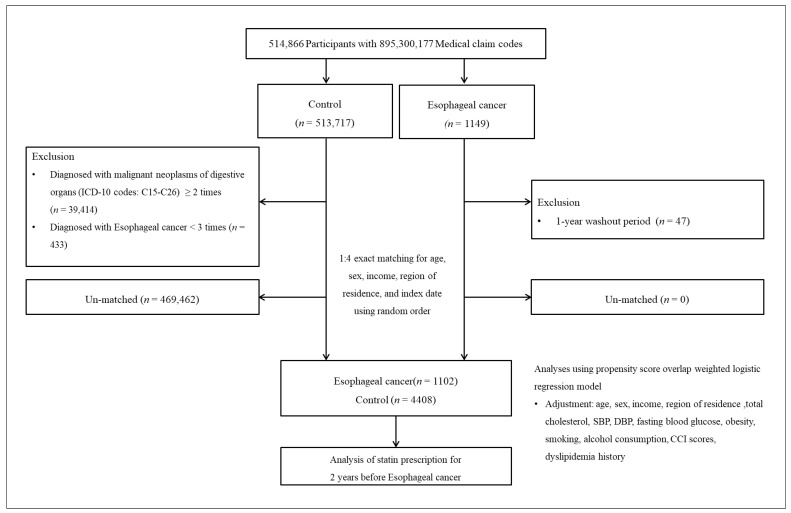
A schematic illustration of the participant selection process used in the present study. Of a total of 514,866 participants, 1102 esophageal cancer participants were matched with 4408 control participants for age, sex, income, and region of residence.

During the matching procedure, 469,462 control participants were removed. Consequently, 1102 esophageal cancer participants and 4408 control participants were enrolled (Figure 1).

### 4.3. Exposure (Statins)

The dates of statin prescription were classified as <180 days, 180 to 545 days, and >545 days for 2 years (730 days) before the diagnosis of esophageal cancer.

### 4.4. Outcome (Esophageal Cancer)

Esophageal cancer was categorized using ICD-10 codes (Malignant neoplasm of esophagus, C15). The participants who visited the clinics ≥3 times with the diagnosis of esophageal cancer were selected. Among them, we divided esophageal cancer participants into three types: those who did not receive medication, those who underwent only surgery, and those who underwent surgery, chemotherapy, or radiotherapy.

### 4.5. Covariates

A total of 10 age groups were categorized. Five income groups were defined based on the national health insurance system. The region of residence was set as urban and rural areas [21]. Tobacco smoking and alcohol consumption were surveyed. Obesity was grouped based on BMI (body mass index, kg/m^2^) measures [22]. Systolic blood pressure (SBP, mmHg), diastolic blood pressure (DBP, mmHg), fasting blood glucose (mg/dL), and total cholesterol (mg/dL) were measured during the health screening procedure.

The comorbidities were quantified using the Charlson Comorbidity Index (CCI) [23]. Esophageal cancer was excluded from the CCI score.

The history of dyslipidemia (ICD-10 code: E78) was defined as the participants who were treated ≥2 times.

### 4.6. Statistical Analyses

We conducted propensity score (PS) overlap weighting. The PS was estimated based on the multivariable logistic regression including all variables. Overlap weighting is calculated between 0 and 1 and achieves exact balance and optimizes precision [24,25,26]. General characteristics were compared between the esophageal cancer and control groups using the standardized difference.

The overlap-weighted odds ratios (ORs) of prescription dates of statins for esophageal cancer were calculated using propensity score overlap-weighted multivariable logistic regression analysis. Crude (unadjusted) and overlap weighted models (adjusted for age, sex, income, region of residence, obesity, smoking, alcohol consumption, systolic blood pressure, diastolic blood pressure, fasting blood glucose, total cholesterol, CCI scores, and dyslipidemia history) were applied. The 95% confidence interval (CI) was analyzed. Additionally, the overlap weighted ORs of prescription dates of statins for mortality in esophageal cancer participants were analyzed. Secondary analyses according to all covariate variables were performed (Tables S1–S6).

Two-tailed analyses were performed, and *p* < 0.05 was set as a statistical significance. The analyses were conducted using SAS version 9.4 (SAS Institute Inc., Cary, NC, USA).

## 5. Conclusions

Long-term statin use was not related to the decreased diagnosis or mortality of esophageal cancer. Both hydrophilic and lipophilic statins did not demonstrate protective effects for the diagnosis or mortality of esophageal cancer in the overall adult Korean population. Only a subset of the study population indicated a lower diagnosis or mortality of esophageal cancer associated with statin use.

## Figures and Tables

**Table 1:** General Characteristics of Participants.

Characteristics	Before Overlap Weighting Adjustment			After Overlap Weighting Adjustment		
	Esophageal Cancer	Control	Standardized Difference	Esophageal Cancer	Control	Standardized Difference
Age (%)			0.00			0.00
40–44	2 (0.18)	8 (0.18)		1 (0.12)	1 (0.12)	
45–49	15 (1.36)	60 (1.36)		7 (1.13)	7 (1.13)	
50–54	59 (5.35)	236 (5.35)		25 (4.37)	25 (4.37)	
55–59	129 (11.71)	516 (11.71)		57 (9.92)	57 (9.92)	
60–64	187 (16.97)	748 (16.97)		86 (14.96)	86 (14.96)	
65–69	236 (21.42)	944 (21.42)		125 (21.73)	125 (21.73)	
70–74	206 (18.69)	824 (18.69)		111 (19.30)	111 (19.30)	
75–79	165 (14.97)	660 (14.97)		100 (17.47)	100 (17.47)	
80–84	86 (7.80)	344 (7.80)		52 (9.11)	52 (9.11)	
85+	17 (1.54)	68 (1.54)		11 (1.88)	11 (1.88)	
Sex (%)			0.00			0.00
Male	1021 (92.65)	4084 (92.65)		528 (91.92)	528 (91.92)	
Female	81 (7.35)	324 (7.35)		46 (8.08)	46 (8.08)	
Income (%)			0.00			0.00
1 (lowest)	185 (16.79)	740 (16.79)		96 (16.75)	96 (16.75)	
2	139 (12.61)	556 (12.61)		72 (12.47)	72 (12.47)	
3	193 (17.51)	772 (17.51)		99 (17.29)	99 (17.29)	
4	231 (20.96)	924 (20.96)		120 (20.94)	120 (20.94)	
5 (highest)	354 (32.12)	1416 (32.12)		187 (32.55)	187 (32.55)	
Region of residence (%)			0.00			0.00
Urban	416 (37.75)	1664 (37.75)		219 (38.05)	219 (38.05)	
Rural	686 (62.25)	2744 (62.25)		356 (61.95)	356 (61.95)	
Obesity † (%)			0.19			0.00
Underweight	78 (7.08)	133 (3.02)		30 (5.25)	30 (5.25)	
Normal	521 (47.28)	1617 (36.68)		254 (44.21)	254 (44.21)	
Overweight	266 (24.14)	1237 (28.06)		147 (25.56)	147 (25.56)	
Obese I	219 (19.87)	1316 (29.85)		132 (23.03)	132 (23.03)	
Obese II	18 (1.63)	105 (2.38)		11 (1.95)	11 (1.95)	
Smoking status (%)			0.28			0.00
Nonsmoker	403 (36.57)	2219 (50.34)		237 (41.25)	237 (41.25)	
Past smoker	281 (25.50)	1139 (25.84)		152 (26.53)	152 (26.53)	
Current smoker	418 (37.93)	1050 (23.82)		185 (32.22)	185 (32.22)	
Alcohol consumption (%)			0.23			0.00
<1 time a week	437 (39.66)	2265 (51.38)		259 (45.15)	259 (45.15)	
≥1 time a week	665 (60.34)	2143 (48.62)		315 (54.85)	315 (54.85)	
SBP (Mean, SD)	129.69 (17.77)	129.82 (16.58)	0.01	129.90 (12.72)	129.90 (6.26)	0.00
DBP (Mean, SD)	78.54 (10.81)	79.15 (10.52)	0.02	78.45 (7.76)	78.45 (3.83)	0.00
Total cholesterol (Mean, SD)	185.25 (39.73)	191.04 (39.05)	0.07	186.81 (28.36)	186.81 (14.01)	0.00
Fasting blood glucose (Mean, SD)	105.27 (30.15)	104.85 (29.78)	0.05	105.77 (20.92)	105.77 (11.96)	0.00
CCI score (Mean, SD)	4.19 (2.31)	1.11 (1.68)	0.71	2.99 (1.19)	2.99 (0.93)	0.00
Dyslipidemia history (%)	407 (36.93)	2237 (50.75)	0.28	242 (42.20)	242 (42.20)	0.00

Abbreviations: CCI, Charlson Comorbidity Index; SBP, Systolic blood pressure; DBP, Diastolic blood pressure; PS, Propensity score. † Obesity (BMI, body mass index, kg/m^2^) was categorized as <18.5 (underweight), ≥18.5 to <23 (normal), ≥23 to <25 (overweight), ≥25 to <30 (obese I), and ≥30 (obese II).

**Table 2:** Crude and overlap propensity score weighted odd ratios of dates of statin prescription for esophageal cancer.

Characteristics	N of Esophageal Cancer	N of Control	Odd Ratios for Esophageal Cancer (95% Confidence Interval)			
	(Exposure/Total, %)	(Exposure/Total, %)	Crude	*p*-Value	Overlap Weighted Model †	*p*-Value
Any statin prescription						
<180 days	914/1102 (82.9)	3618/4408 (82.1)	1		1	
180 to 545 days	65/1102 (5.9)	270/4408 (6.1)	0.95 (0.72–1.26)	0.736	1.30 (1.03–1.65)	0.027 *
>545 days	123/1102 (11.2)	520/4408 (11.8)	0.94 (0.76–1.15)	0.538	1.29 (1.08–1.55)	0.006 *
Hydrophilic statin prescription						
<180 days	1043/1102 (94.7)	4178/4408 (94.8)	1		1	
180 to 545 days	21/1102 (1.9)	105/4408 (2.4)	0.80 (0.50–1.29)	0.359	1.04 (0.73–1.49)	0.818
>545 days	38/1102 (3.5)	125/4408 (2.8)	1.22 (0.84–1.76)	0.296	1.45 (1.08–1.94)	0.013 *
Lipophilic statin prescription						
<180 days	968/1102 (87.8)	3820/4408 (86.7)	1		1	
180 to 545 days	53/1102 (4.8)	225/4408 (5.1)	0.93 (0.68–1.26)	0.642	1.40 (1.08–1.82)	0.012 *
>545 days	81/1102 (7.4)	363/4408 (8.2)	0.88 (0.69–1.13)	0.321	1.15 (0.93–1.41)	0.187

Abbreviations: CCI, Charlson Comorbidity Index; SBP, Systolic blood pressure; DBP, Diastolic blood pressure; * Significance at *p* < 0.05; † Adjusted for age, sex, income, region of residence, SBP, DBP, fasting blood glucose, total cholesterol, obesity, smoking, alcohol consumption, dyslipidemia history, and CCI scores.

**Table 3:** General Characteristics of Esophageal Cancer Participants.

Characteristics	Before Overlap Weighting Adjustment			After Overlap Weighting Adjustment		
	Dead Participants	Survived Participants	Standardized Difference	Dead Participants	Survived Participants	Standardized Difference
Age (%)			0.31			0.00
40–44	0.15	0.23		0.10	0.10	
45–49	1.05	1.84		0.94	0.94	
50–54	4.79	6.22		4.51	4.51	
55–59	10.03	14.29		11.78	11.78	
60–64	13.77	21.89		18.86	18.86	
65–69	20.36	23.04		21.84	21.84	
70–74	20.51	15.90		18.49	18.49	
75–79	17.22	11.52		15.36	15.36	
80–84	10.18	4.15		6.62	6.62	
85+	1.95	0.92		1.51	1.51	
Sex (%)			0.17			0.00
Male	94.46	89.86		92.66	92.66	
Female	5.54	10.14		7.34	7.34	
Income (%)			0.10			0.00
1 (lowest)	18.26	14.52		16.94	16.94	
2	14.22	10.14		10.55	10.55	
3	16.47	19.12		17.72	17.72	
4	20.06	22.35		22.46	22.46	
5 (highest)	30.99	33.87		32.33	32.33	
Region of residence (%)			0.13			0.00
Urban	35.18	41.71		39.26	39.26	
Rural	64.82	58.29		60.74	60.74	
Obesity † (%)			0.21			0.00
Underweight	9.13	3.92		6.3	6.3	
Normal	52.69	38.94		46.83	46.83	
Overweight	21.56	28.11		23.66	23.66	
Obese I	15.42	26.73		21.2	21.2	
Obese II	1.2	2.3		2.01	2.01	
Smoking status (%)			0.03			0.00
Nonsmoker	36.08	37.33		37.95	37.95	
Past smoker	21.41	31.8		26.39	26.39	
Current smoker	42.51	30.88		35.65	35.65	
Alcohol consumption (%)			0.08			0.00
<1 time a week	41.17	37.33		40.01	40.01	
≥1 time a week	58.83	62.67		59.99	59.99	
SBP (Mean, SD)	130.80 (18.16)	127.98 (17.04)	0.16	129.48 (9.09)	129.48 (11.75)	0.00
DBP (Mean, SD)	79.02 (10.76)	77.80 (10.86)	0.11	78.16 (5.51)	78.16 (7.40)	0.00
Total cholesterol (Mean, SD)	184.64 (39.38)	186.19 (40.29)	0.04	105.94 (16.63)	105.94 (18.35)	0.00
Fasting blood glucose (Mean, SD)	105.55 (32.20)	104.84 (26.72)	0.02	184.17 (20.67)	184.17 (26.96)	0.00
CCI score (Mean, SD)	4.76 (2.38)	3.31 (1.90)	0.67	3.94 (1.10)	3.94 (1.36)	0.00
Dyslipidemia history (%)	27.4	51.61	0.51	39.73	39.73	0.00
Treatment type (%)			0.55			0.00
No treatment	30.54	39.17		32.5	32.5	
Only surgery	8.83	23.96		16.46	16.46	
Surgery or RT or CT	60.63	36.87		51.03	51.03	

Abbreviations: CCI, Charlson Comorbidity Index; SBP, Systolic blood pressure; DBP, Diastolic blood pressure; PS, Propensity score; RT, Radiotherapy; CT, Chemotherapy. † Obesity (BMI, body mass index, kg/m^2^) was categorized as <18.5 (underweight), ≥18.5 to <23 (normal), ≥23 to <25 (overweight), ≥25 to <30 (obese I), and ≥30 (obese II).

**Table 4:** Crude and overlap propensity score weighted odd ratios of dates of statin prescription for mortality in esophageal cancer participants.

Characteristics	Dead Participants	Survived Participants	Odd Ratios for Mortality (95% Confidence Interval)			
	(Exposure/Total, %)	(Exposure/Total, %)	Crude	*p*-Value	Overlap Weighted Model †	*p*-Value
Any statin prescription						
<180 days	574/434 (85.9)	340/668 (78.3)	1		1	
180 to 545 days	33/434 (4.9)	32/668 (7.4)	0.61 (0.37–1.01)	0.056	0.99 (0.58–1.70)	0.975
>545 days	61/434 (9.1)	62/668 (14.3)	0.58 (0.40–0.85)	0.005	1.03 (0.68–1.55)	0.739
Hydrophilic statin prescription						
<180 days	644/434 (96.4)	399/668 (91.9)	1		1	
180 to 545 days	12/434 (1.8)	09/668 (2.1)	0.83 (0.34–1.98)	0.668	1.55 (0.54–4.41)	0.413
>545 days	12/434 (1.8)	26/668 (6.0)	0.29 (0.14–0.57)	<0.001 *	0.66 (0.34–1.26)	0.206
Lipophilic statin prescription						
<180 days	594/434 (88.9)	374/668 (86.2)	1		1	
180 to 545 days	29/434 (4.3)	24/668 (5.5)	0.76 (0.44–1.33)	0.335	1.26 (0.72–2.21)	0.423
>545 days	45/434 (6.7)	36/668 (8.3)	0.79 (0.50–1.24)	0.304	1.21 (0.76–1.94)	0.419

Abbreviations: CCI, Charlson Comorbidity Index; SBP, Systolic blood pressure; DBP, Diastolic blood pressure; * Significance at *p* < 0.05; † Adjusted for age, sex, income, region of residence, SBP, DBP, fasting blood glucose, total cholesterol, obesity, smoking, alcohol consumption, dyslipidemia history, CCI scores, and treatment type.

## Data Availability

Data is contained within the article and Supplementary Material.

## Supplementary Materials

The following supporting information can be downloaded at: https://www.mdpi.com/article/10.3390/ph16060900/s1, Table S1: Crude and overlap propensity score weighted odd ratios of dates of any statin prescription for esophageal cancer, Table S2: Crude and overlap propensity score weighted odd ratios of dates of hydrophilic statin prescription for esophageal cancer. Table S3: Crude and overlap propensity score weighted odd ratios of dates of lipophilic statin prescription for esophageal cancer. Table S4: Crude and overlap propensity score weighted odd ratios of dates of statin prescription for mortality in esophageal cancer participants. Table S5: Crude and overlap propensity score weighted odd ratios of dates of hydrophilic prescription for mortality in esophageal cancer participants. Table S6: Crude and overlap propensity score weighted odd ratios of dates of lipophilic statin prescription for mortality in esophageal cancer. Figure S1: Crude and overlap propensity score weighted odd ratios of dates of any statin prescription for esophageal cancer, Figure S2: Crude and overlap propensity score weighted odd ratios of dates of hydrophilic statin prescription for esophageal cancer. Figure S3: Crude and overlap propensity score weighted odd ratios of dates of lipophilic statin prescription for esophageal cancer. Figure S4: Crude and overlap propensity score weighted odd ratios of dates of statin prescription for mortality in esophageal cancer participants. Figure S5: Crude and overlap propensity score weighted odd ratios of dates of hydrophilic prescription for mortality in esophageal cancer participants. Figure S6: Crude and overlap propensity score weighted odd ratios of dates of lipophilic statin prescription for mortality in esophageal cancer.
