# Supplementary material for: Relation of Statin Use with Esophageal Cancer
Source: Pharmaceuticals (Basel). 2023 Jun 19;16(6):900. doi: 10.3390/ph16060900 (PMC10305244; doi:10.3390/ph16060900)
Supplement: Supplementary file 1 [file pharmaceuticals-16-00900-s001.zip › table S1.pdf]

S1 table Crude and overlap propensity score weighted odd ratios of dates of any statin prescription for esophageal cancer.

Characteristics	N of esophageal cancer	N of Control	Odd ratios for esophageal cancer (95% confidence interval)			
	(exposure/total, %)	(exposure/total, %)	Crude	P-value	Overlap weighted model †	P-value
Age < 65 years old (n=1,960)						
< 180 days	342/392 (87.2)	1,361/1,568 (86.8)	1		1	
180 to 545 days	25/392 (6.4)	85/1,568 (5.4)	1.17 (0.74-1.86)	0.504	2.28 (1.51-3.44)	<0.001*
> 545 days	25/392 (6.4)	122/1,568 (7.8)	0.82 (0.52-1.27)	0.371	0.83 (0.57-1.20)	0.327
Age ≥ 65 years old (n=3,550)						
< 180 days	572/710 (80.6)	2,257/2,840 (79.5)	1		1	
180 to 545 days	40/710 (5.6)	185/2,840 (6.5)	0.85 (0.60-1.22)	0.379	1.07 (0.80-1.44)	0.660
> 545 days	98/710 (13.8)	398/2,840 (14.0)	0.97 (0.76-1.23)	0.813	1.35 (1.09-1.68)	0.007*
Male (n=5,105)						
< 180 days	852/1,021 (83.4)	3,369/4,084 (82.5)	1		1	
180 to 545 days	55/1,021 (5.4)	238/4,084 (5.8)	0.91 (0.68-1.24)	0.559	1.30 (1.01-1.67)	0.043*
> 545 days	114/1,021 (11.2)	477/4,084 (11.7)	0.95 (0.76-1.17)	0.611	1.34 (1.11-1.61)	0.002*
Female (n=405)						
< 180 days	62/81 (76.5)	249/324 (76.9)	1		1	
180 to 545 days	10/81 (12.3)	32/324 (9.9)	1.26 (0.59-2.69)	0.559	1.17 (0.56-2.44)	0.668
> 545 days	9/81 (11.1)	43/324 (13.3)	0.84 (0.39-1.82)	0.659	0.86 (0.39-1.89)	0.711
Low income groups (n=2,585)						

< 180 days	440/517 (85.1)	1,730/2,068 (83.7)	1		1	
180 to 545 days	30/517 (5.8)	119/2,068 (5.8)	0.99 (0.66-1.50)	0.967	1.65 (1.14-2.38)	0.008*
> 545 days	47/517 (9.1)	219/2,068 (10.6)	0.84 (0.61-1.18)	0.316	1.44 (1.07-1.95)	0.016*
High income groups (n=2,925)						
< 180 days	474/585 (81.0)	1,888/2,340 (80.7)	1		1	
180 to 545 days	35/585 (6.0)	151/2,340 (6.5)	0.92 (0.63-1.35)	0.681	1.11 (0.82-1.52)	0.493
> 545 days	76/585 (13.0)	301/2,340 (12.9)	1.01 (0.77-1.32)	0.967	1.19 (0.95-1.50)	0.130
Urban residents (n=2,080)						
< 180 days	332/416 (79.8)	1,343/1,664 (80.7)	1		1	
180 to 545 days	25/416 (6.0)	103/1,664 (6.2)	0.98 (0.62-1.54)	0.937	1.14 (0.77-1.67)	0.519
> 545 days	59/416 (14.2)	218/1,664 (13.1)	1.09 (0.80-1.50)	0.569	1.57 (1.18-2.07)	0.002*
Rural residents (n=3,430)						
< 180 days	582/686 (84.8)	2,275/2,744 (82.9)	1		1	
180 to 545 days	40/686 (5.8)	167/2,744 (6.1)	0.94 (0.66-1.34)	0.718	1.48 (1.10-2.00)	0.010*
> 545 days	64/686 (9.3)	302/2,744 (11.0)	0.83 (0.62-1.10)	0.195	1.14 (0.90-1.45)	0.281
Underweight (n=211)						
< 180 days	77/78 (98.7)	120/133 (90.2)	1		1	
180 to 545 days	1/78 (1.3)	3/133 (2.3)	0.52 (0.05-5.08)	0.574	0.01 (0.00-15.6)	0.236
> 545 days	0/78 (0.0)	10/133 (7.5)	N/A		N/A	
Normal weight (n=2,138)						

< 180 days	587/699 (84.0)	1,781/2,189 (81.4)	1		1	
180 to 545 days	37/699 (5.3)	135/2,189 (6.2)	1.19 (0.76-1.84)	0.450	1.52 (1.01-2.27)	0.043*
> 545 days	75/699 (10.7)	273/2,189 (12.5)	0.91 (0.63-1.31)	0.610	1.20 (0.86-1.67)	0.277
Overweight (n=1,503)						
< 180 days	451/521 (86.6)	1,401/1,617 (86.6)	1		1	
180 to 545 days	29/521 (5.6)	76/1,617 (4.7)	1.28 (0.77-2.11)	0.343	1.77 (1.16-2.69)	0.008*
> 545 days	41/521 (7.9)	140/1,617 (8.7)	1.11 (0.75-1.65)	0.588	1.34 (0.96-1.86)	0.084
Obese (n=1,658)						
< 180 days	209/266 (78.6)	1,003/1,237 (81.1)	1		1	
180 to 545 days	21/266 (7.9)	79/1,237 (6.4)	0.77 (0.43-1.38)	0.382	0.94 (0.62-1.42)	0.768
> 545 days	36/266 (13.5)	155/1,237 (12.5)	1.32 (0.93-1.89)	0.124	1.49 (1.11-1.98)	0.007*
Non-smoker (n=2,622)						
< 180 days	177/237 (74.7)	1,094/1,421 (77.0)	1		1	
180 to 545 days	14/237 (5.9)	112/1,421 (7.9)	0.86 (0.75-0.98)	0.022*	0.86 (0.78-0.96)	0.005*
> 545 days	46/237 (19.4)	215/1,421 (15.1)	1.00 (0.89-1.13)	0.988	1.05 (0.95-1.16)	0.343
Past and current smoker (n=2,808)						
< 180 days	587/699 (84.0)	1,781/2,189 (81.4)	1		1	
180 to 545 days	37/699 (5.3)	135/2,189 (6.2)	0.95 (0.81-1.11)	0.490	0.91 (0.79-1.04)	0.148
> 545 days	75/699 (10.7)	273/2,189 (12.5)	0.87 (0.75-1.00)	0.055	0.84 (0.73-0.95)	0.006*
Alcohol consumption <1 time a week (n=2,702)						

< 180 days	346/437 (79.2)	1,860/2,265 (82.1)	1		1	
180 to 545 days	29/437 (6.6)	143/2,265 (6.3)	0.89 (0.78-1.01)	0.072	0.90 (0.81-1.01)	0.062
> 545 days	62/437 (14.2)	262/2,265 (11.6)	0.87 (0.76-0.98)	0.027*	0.92 (0.83-1.02)	0.100
Alcohol consumption ≥1 time a week (n=2,808)						
< 180 days	568/665 (85.4)	1,758/2,143 (82.0)	1		1	
180 to 545 days	36/665 (5.4)	127/2,143 (5.9)	0.90 (0.77-1.04)	0.156	0.84 (0.74-0.96)	0.008*
> 545 days	61/665 (9.2)	258/2,143 (12.0)	1.05 (0.91-1.21)	0.494	1.00 (0.89-1.14)	0.964
SBP < 140 mmHg and DBP < 90 mmHg (n=3,811)						
< 180 days	642/776 (82.7)	2,492/3,035 (82.1)	1		1	
180 to 545 days	46/776 (5.9)	186/3,035 (6.1)	0.85 (0.75-0.96)	0.008*	0.86 (0.77-0.95)	0.002*
> 545 days	88/776 (11.3)	357/3,035 (11.8)	0.93 (0.83-1.04)	0.202	0.93 (0.85-1.03)	0.160
SBP ≥ 140 mmHg or DBP ≥ 90 mmHg (n=1,699)						
< 180 days	272/326 (83.4)	1,126/1,373 (82.0)	1		1	
180 to 545 days	19/326 (5.8)	84/1,373 (6.1)	0.99 (0.83-1.17)	0.870	0.94 (0.81-1.08)	0.377
> 545 days	35/326 (10.7)	163/1,373 (11.9)	0.97 (0.82-1.16)	0.765	1.02 (0.88-1.18)	0.831
Fasting blood glucose < 100 mg/dL (n=2,944)						
< 180 days	504/585 (86.2)	2,044/2,359 (86.6)	1		1	
180 to 545 days	32/585 (5.5)	103/2,359 (4.4)	0.88 (0.77-1.02)	0.097	0.94 (0.83-1.05)	0.278
> 545 days	49/585 (8.4)	212/2,359 (9.0)	0.95 (0.82-1.10)	0.484	0.97 (0.86-1.09)	0.588
Fasting blood glucose ≥ 100 mg/dL (n=2,566)						

< 180 days	410/517 (79.3)	1,574/2,049 (76.8)	1		1	
180 to 545 days	33/517 (6.4)	167/2,049 (8.2)	0.88 (0.76-1.01)	0.067	0.84 (0.75-0.94)	0.003*
> 545 days	74/517 (14.3)	308/2,049 (15.0)	0.92 (0.81-1.04)	0.167	0.94 (0.85-1.05)	0.278
Total cholesterol < 200 mg/dL (n=3,415)						
< 180 days	587/719 (81.6)	2,129/2,696 (79.0)	1		1	
180 to 545 days	36/719 (5.0)	145/2,696 (5.4)	0.98 (0.86-1.13)	0.797	0.92 (0.82-1.03)	0.139
> 545 days	96/719 (13.4)	422/2,696 (15.7)	0.95 (0.85-1.06)	0.351	0.96 (0.87-1.06)	0.459
Total cholesterol ≥ 200 mg/dL (n=2,095)						
< 180 days	327/383 (85.4)	1,489/1,712 (87.0)	1		1	
180 to 545 days	29/383 (7.6)	125/1,712 (7.3)	0.81 (0.70-0.94)	0.005*	0.83 (0.74-0.94)	0.002*
> 545 days	27/383 (7.0)	98/1,712 (5.7)	0.85 (0.71-1.03)	0.100	0.86 (0.74-1.00)	0.050
CCI scores = 0 (n=2,351)						
< 180 days	22/24 (91.7)	1,971/2,327 (84.7)	1		1	
180 to 545 days	1/24 (4.2)	132/2,327 (5.7)	0.68 (0.09-5.07)	0.506	0.54 (0.29-1.01)	0.053
> 545 days	1/24 (4.2)	224/2,327 (9.6)	0.40 (0.05-2.98)	0.511	0.38 (0.21-0.71)	0.002*
CCI scores = 1 (n=909)						
< 180 days	18/25 (72.0)	699/884 (79.1)	1		1	
180 to 545 days	4/25 (16.0)	52/884 (5.9)	2.99 (0.98-9.15)	0.004*	5.17 (2.58-10.3)	<0.001*
> 545 days	3/25 (12.0)	133/884 (15.0)	0.88 (0.25-3.02)	0.130	1.07 (0.57-2.01)	0.831
CCI scores ≥ 2 (n=2,250)						

< 180 days	874/1,053 (83.0)	948/1,197 (79.2)	1		1	
180 to 545 days	60/1,053 (5.7)	86/1,197 (7.2)	0.76 (0.54-1.07)	<0.001*	1.18 (0.80-1.76)	0.406
> 545 days	119/1,053 (11.3)	163/1,197 (13.6)	0.79 (0.61-1.02)	<0.001*	1.33 (0.99-1.80)	0.060
Non dyslipidemia history (n=2,866)						
< 180 days	671/695 (96.5)	2,090/2,171 (96.3)	1		1	
180 to 545 days	9/695 (1.3)	23/2,171 (1.1)	0.79 (0.59-1.04)	0.097	0.65 (0.52-0.80)	<0.001*
> 545 days	15/695 (2.2)	58/2,171 (2.7)	0.88 (0.66-1.18)	0.394	0.73 (0.59-0.92)	0.007*
Dyslipidemia history (n=2,644)						
< 180 days	243/407 (59.7)	1,528/2,237 (68.3)	1		1	
180 to 545 days	56/407 (13.8)	247/2,237 (11.0)	1.04 (0.93-1.16)	0.500	0.93 (0.86-1.02)	0.124
> 545 days	108/407 (26.5)	462/2,237 (20.7)	1.09 (0.98-1.22)	0.098	0.96 (0.89-1.05)	0.410

Abbreviations: CCI, Charlson Comorbidity Index; SBP, Systolic blood pressure; DBP, Diastolic blood pressure;

* Significance at P < 0.05

† Adjusted for age, sex, income, region of residence, SBP, DBP, fasting blood glucose, total cholesterol, obesity, smoking, alcohol consumption, dyslipidemia history, and CCI scores.
