# Supplementary material for: Relation of Statin Use with Esophageal Cancer
Source: Pharmaceuticals (Basel). 2023 Jun 19;16(6):900. doi: 10.3390/ph16060900 (PMC10305244; doi:10.3390/ph16060900)
Supplement: Supplementary file 1 [file pharmaceuticals-16-00900-s001.zip › table S2.pdf]

S2 table Crude and overlap propensity score weighted odd ratios of dates of hydrophilic statin prescription for esophageal cancer.

Characteristics	N of esophageal cancer	N of Control	Odd ratios for esophageal cancer (95% confidence interval)			
	(exposure/total, %)	(exposure/total, %)	Crude	P-value	Overlap weighted model †	P-value
Age < 65 years old (n=1,960)						
< 180 days	376/392 (95.9)	1,517/1,568 (96.7)	1		1	
180 to 545 days	6/392 (1.5)	24/1,568 (1.5)	1.01 (0.41-2.49)	0.985	2.65 (1.24-5.68)	0.012*
> 545 days	10/392 (2.6)	27/1,568 (1.7)	1.49 (0.72-3.12)	0.283	0.93 (0.51-1.70)	0.806
Age ≥ 65 years old (n=3,550)						
< 180 days	667/710 (93.9)	2,661/2,840 (93.7)	1		1	
180 to 545 days	15/710 (2.1)	81/2,840 (2.9)	0.74 (0.42-1.29)	0.288	0.86 (0.57-1.31)	0.485
> 545 days	28/710 (3.9)	98/2,840 (3.5)	1.14 (0.74-1.75)	0.549	1.49 (1.05-2.10)	0.026*
Male (n=5,105)						
< 180 days	968/1,021 (94.8)	3,885/4,084 (95.1)	1		1	
180 to 545 days	17/1,021 (1.7)	90/4,084 (2.2)	0.76 (0.45-1.28)	0.300	0.98 (0.66-1.45)	0.920
> 545 days	36/1,021 (3.5)	109/4,084 (2.7)	1.33 (0.90-1.94)	0.149	1.60 (1.18-2.16)	0.002*
Female (n=405)						
< 180 days	75/81 (92.6)	293/324 (90.4)	1		1	
180 to 545 days	4/81 (4.9)	15/324 (4.6)	1.04 (0.34-3.23)	0.944	1.10 (0.43-2.85)	0.837
> 545 days	2/81 (2.5)	16/324 (4.9)	0.49 (0.11-2.17)	0.346	0.37 (0.09-1.51)	0.167
Low income groups (n=2,585)						

< 180 days	489/517 (94.6)	1,975/2,068 (95.5)	1		1	
180 to 545 days	11/517 (2.1)	40/2,068 (1.9)	1.11 (0.57-2.18)	0.760	1.85 (1.07-3.18)	0.027*
> 545 days	17/517 (3.3)	53/2,068 (2.6)	1.30 (0.74-2.26)	0.361	2.07 (1.29-3.34)	0.003*
High income groups (n=2,925)						
< 180 days	554/585 (94.7)	2,203/2,340 (94.1)	1		1	
180 to 545 days	10/585 (1.7)	65/2,340 (2.8)	0.61 (0.31-1.20)	0.152	0.68 (0.42-1.11)	0.122
> 545 days	21/585 (3.6)	72/2,340 (3.1)	1.16 (0.71-1.90)	0.557	1.14 (0.78-1.66)	0.500
Urban residents (n=2,080)						
< 180 days	388/416 (93.3)	1,566/1,664 (94.1)	1		1	
180 to 545 days	7/416 (1.7)	45/1,664 (2.7)	0.63 (0.28-1.40)	0.257	0.85 (0.47-1.55)	0.603
> 545 days	21/416 (5.0)	53/1,664 (3.2)	1.60 (0.95-2.68)	0.075	2.39 (1.50-3.81)	<0.001*
Rural residents (n=3,430)						
< 180 days	655/686 (95.5)	2,612/2,744 (95.2)	1		1	
180 to 545 days	14/686 (2.0)	60/2,744 (2.2)	0.93 (0.52-1.68)	0.810	1.27 (0.81-1.99)	0.306
> 545 days	17/686 (2.5)	72/2,744 (2.6)	0.94 (0.55-1.61)	0.826	1.03 (0.70-1.52)	0.879
Underweight (n=211)						
< 180 days	77/78 (98.7)	128/133 (96.2)	1		1	
180 to 545 days	1/78 (1.3)	2/133 (1.5)	0.83 (0.07-9.32)	0.881	0.02 (0.00-26.0)	0.295
> 545 days	0/78 (0.0)	3/133 (2.3)	N/A		N/A	
Normal weight (n=2,138)						

< 180 days	497/521 (95.4)	1,558/1,617 (96.4)	1		1	
180 to 545 days	11/521 (2.1)	23/1,617 (1.4)	1.50 (0.73-3.10)	0.274	1.88 (1.02-3.47)	0.043*
> 545 days	13/521 (2.5)	36/1,617 (2.2)	1.13 (0.60-2.15)	0.705	1.66 (0.94-2.91)	0.081
Overweight (n=1,503)						
< 180 days	250/266 (94.0)	1,174/1,237 (94.9)	1		1	
180 to 545 days	5/266 (1.9)	27/1,237 (2.2)	0.87 (0.33-2.28)	0.776	1.15 (0.58-2.30)	0.682
> 545 days	11/266 (4.1)	36/1,237 (2.9)	1.44 (0.72-2.86)	0.304	2.13 (1.22-3.72)	0.008*
Obese (n=1,658)						
< 180 days	219/237 (92.4)	1,318/1,421 (92.8)	1		1	
180 to 545 days	4/237 (1.7)	53/1,421 (3.7)	0.45 (0.16-1.27)	0.132	0.49 (0.27-0.91)	0.023*
> 545 days	14/237 (5.9)	50/1,421 (3.5)	1.69 (0.92-3.10)	0.093	1.10 (0.72-1.68)	0.657
Non-smoker (n=2,622)						
< 180 days	381/403 (94.5)	2,115/2,219 (95.3)	1		1	
180 to 545 days	13/403 (3.2)	45/2,219 (2.0)	1.60 (0.86-3.00)	0.140	1.99 (1.22-3.22)	0.006*
> 545 days	9/403 (2.2)	59/2,219 (2.7)	0.85 (0.42-1.72)	0.646	0.79 (0.49-1.28)	0.345
Past and current smoker (n=2,808)						
< 180 days	662/699 (94.7)	2,063/2,189 (94.2)	1		1	
180 to 545 days	8/699 (1.1)	60/2,189 (2.7)	0.42 (0.20-0.87)	0.021*	0.47 (0.26-0.85)	0.012*
> 545 days	29/699 (4.1)	66/2,189 (3.0)	1.37 (0.88-2.14)	0.167	1.95 (1.33-2.88)	0.001*
Alcohol consumption <1 time a week (n=2,702)						

< 180 days	412/437 (94.3)	2,149/2,265 (94.9)	1		1	
180 to 545 days	9/437 (2.1)	54/2,265 (2.4)	0.87 (0.43-1.77)	0.701	1.08 (0.64-1.82)	0.785
> 545 days	16/437 (3.7)	62/2,265 (2.7)	1.35 (0.77-2.36)	0.298	1.58 (1.02-2.46)	0.042*
Alcohol consumption ≥1 time a week (n=2,808)						
< 180 days	631/665 (94.9)	2,029/2,143 (94.7)	1		1	
180 to 545 days	12/665 (1.8)	51/2,143 (2.4)	0.76 (0.40-1.43)	0.390	1.02 (0.62-1.66)	0.952
> 545 days	22/665 (3.3)	63/2,143 (2.9)	1.12 (0.69-1.84)	0.645	1.36 (0.92-2.03)	0.127
SBP < 140 mmHg and DBP < 90 mmHg (n=3,811)						
< 180 days	726/776 (93.6)	2,869/3,035 (94.5)	1		1	
180 to 545 days	18/776 (2.3)	79/3,035 (2.6)	0.90 (0.54-1.51)	0.692	1.23 (0.83-1.83)	0.308
> 545 days	32/776 (4.1)	87/3,035 (2.9)	1.45 (0.96-2.20)	0.076	1.78 (1.27-2.49)	0.001*
SBP ≥ 140 mmHg or DBP ≥ 90 mmHg (n=1,699)						
< 180 days	317/326 (97.2)	1,309/1,373 (95.3)	1		1	
180 to 545 days	3/326 (0.9)	26/1,373 (1.9)	0.48 (0.14-1.58)	0.227	0.55 (0.23-1.35)	0.192
> 545 days	6/326 (1.8)	38/1,373 (2.8)	0.65 (0.27-1.56)	0.335	0.68 (0.35-1.32)	0.257
Fasting blood glucose < 100 mg/dL (n=2,944)						
< 180 days	563/585 (96.2)	2,273/2,359 (96.4)	1		1	
180 to 545 days	7/585 (1.2)	37/2,359 (1.6)	0.76 (0.34-1.72)	0.516	0.82 (0.45-1.50)	0.523
> 545 days	15/585 (2.6)	49/2,359 (2.1)	1.24 (0.69-2.22)	0.478	1.50 (0.92-2.43)	0.103
Fasting blood glucose ≥ 100 mg/dL (n=2,566)						

< 180 days	480/517 (92.8)	1,905/2,049 (93.0)	1		1	
180 to 545 days	14/517 (2.7)	68/2,049 (3.3)	0.82 (0.46-1.47)	0.498	1.12 (0.72-1.76)	0.607
> 545 days	23/517 (4.4)	76/2,049 (3.7)	1.20 (0.75-1.94)	0.452	1.46 (1.01-2.12)	0.046*
Total cholesterol < 200 mg/dL (n=3,415)						
< 180 days	671/719 (93.3)	2,524/2,696 (93.6)	1		1	
180 to 545 days	16/719 (2.2)	67/2,696 (2.5)	0.90 (0.52-1.56)	0.703	1.33 (0.85-2.07)	0.209
> 545 days	32/719 (4.5)	105/2,696 (3.9)	1.15 (0.76-1.72)	0.508	1.56 (1.11-2.20)	0.011*
Total cholesterol ≥ 200 mg/dL (n=2,095)						
< 180 days	372/383 (97.1)	1,654/1,712 (96.6)	1		1	
180 to 545 days	5/383 (1.3)	38/1,712 (2.2)	0.59 (0.23-1.50)	0.263	0.60 (0.32-1.14)	0.120
> 545 days	6/383 (1.6)	20/1,712 (1.2)	1.33 (0.53-3.34)	0.539	1.11 (0.60-2.07)	0.734
CCI scores = 0 (n=2,351)						
< 180 days	23/24 (95.8)	2,232/2,327 (95.9)	1		1	
180 to 545 days	0/24 (0.0)	47/2,327 (2.0)	N/A	0.984	N/A	0.975
> 545 days	1/24 (4.2)	48/2,327 (2.1)	2.02 (0.27-15.3)	0.495	2.52 (1.27-4.98)	0.008*
CCI scores = 1 (n=909)						
< 180 days	22/25 (88.0)	821/884 (92.9)	1		1	
180 to 545 days	3/25 (12.0)	28/884 (3.2)	4.00 (1.13-14.1)	0.032*	5.57 (2.50-12.4)	<0.001*
> 545 days	0/25 (0.0)	35/884 (4.0)	N/A		N/A	
CCI scores ≥ 2 (n=2,250)						

< 180 days	998/1,053 (94.8)	1,125/1,197 (94.0)	1		1	
180 to 545 days	18/1,053 (1.7)	30/1,197 (2.5)	0.68 (0.37-1.22)	0.194	0.90 (0.49-1.67)	0.747
> 545 days	37/1,053 (3.5)	42/1,197 (3.5)	0.99 (0.63-1.56)	0.976	1.35 (0.84-2.19)	0.217
Non dyslipidemia history (n=2,866)						
< 180 days	688/695 (99.0)	2,144/2,171 (98.8)	1		1	
180 to 545 days	4/695 (0.6)	10/2,171 (0.5)	1.25 (0.39-3.99)	0.710	1.23 (0.53-2.88)	0.631
> 545 days	3/695 (0.4)	17/2,171 (0.8)	0.55 (0.16-1.88)	0.341	0.75 (0.28-2.01)	0.572
Dyslipidemia history (n=2,644)						
< 180 days	355/407 (87.2)	2,034/2,237 (90.9)	1		1	
180 to 545 days	17/407 (4.2)	95/2,237 (4.2)	1.03 (0.60-1.74)	0.926	1.02 (0.70-1.49)	0.910
> 545 days	35/407 (8.6)	108/2,237 (4.8)	1.86 (1.25-2.76)	0.002*	1.58 (1.17-2.11)	0.003*

Abbreviations: CCI, Charlson Comorbidity Index; SBP, Systolic blood pressure; DBP, Diastolic blood pressure;

* Significance at P < 0.05

† Adjusted for age, sex, income, region of residence, SBP, DBP, fasting blood glucose, total cholesterol, obesity, smoking, alcohol consumption, dyslipidemia history, and CCI scores.
