# Supplementary material for: Relation of Statin Use with Esophageal Cancer
Source: Pharmaceuticals (Basel). 2023 Jun 19;16(6):900. doi: 10.3390/ph16060900 (PMC10305244; doi:10.3390/ph16060900)
Supplement: Supplementary file 1 [file pharmaceuticals-16-00900-s001.zip › table S3.pdf]

S3 table Crude and overlap propensity score weighted odd ratios of dates of lipophilic statin prescription for esophageal cancer.

Characteristics	N of esophageal cancer	N of Control	Odd ratios for esophageal cancer (95% confidence interval)			
	(exposure/total, %)	(exposure/total, %)	Crude	P-value	Overlap weighted model †	P-value
Age < 65 years old (n=1,960)						
< 180 days	357/392 (91.1)	1,408/1,568 (89.8)	1		1	
180 to 545 days	20/392 (5.1)	73/1,568 (4.7)	1.08 (0.65-1.80)	0.765	2.23 (1.42-3.50)	0.001*
> 545 days	15/392 (3.8)	87/1,568 (5.5)	0.68 (0.39-1.19)	0.178	0.78 (0.50-1.21)	0.271
Age ≥ 65 years old (n=3,550)						
< 180 days	611/710 (86.1)	2,412/2,840 (84.9)	1		1	
180 to 545 days	33/710 (4.6)	152/2,840 (5.4)	0.86 (0.58-1.26)	0.434	1.17 (0.84-1.63)	0.367
> 545 days	66/710 (9.3)	276/2,840 (9.7)	0.94 (0.71-1.25)	0.690	1.21 (0.95-1.54)	0.125
Male (n=5,105)						
< 180 days	901/1,021 (88.2)	3,543/4,084 (86.8)	1		1	
180 to 545 days	46/1,021 (4.5)	202/4,084 (4.9)	0.90 (0.64-1.24)	0.510	1.38 (1.04-1.82)	0.024*
> 545 days	74/1,021 (7.2)	339/4,084 (8.3)	0.86 (0.66-1.12)	0.253	1.14 (0.92-1.41)	0.227
Female (n=405)						
< 180 days	67/81 (82.7)	277/324 (85.5)	1		1	
180 to 545 days	7/81 (8.6)	23/324 (7.1)	1.26 (0.52-3.06)	0.612	1.53 (0.62-3.75)	0.352
> 545 days	7/81 (8.6)	24/324 (7.4)	1.21 (0.50-2.92)	0.678	1.31 (0.55-3.10)	0.543
Low income groups (n=2,585)						

< 180 days	465/517 (89.9)	1,810/2,068 (87.5)	1		1	
180 to 545 days	25/517 (4.8)	107/2,068 (5.2)	0.91 (0.58-1.42)	0.677	1.58 (1.04-2.40)	0.031*
> 545 days	27/517 (5.2)	151/2,068 (7.3)	0.70 (0.46-1.06)	0.093	0.99 (0.69-1.41)	0.943
High income groups (n=2,925)						
< 180 days	503/585 (86.0)	2,010/2,340 (85.9)	1		1	
180 to 545 days	28/585 (4.8)	118/2,340 (5.0)	0.95 (0.62-1.45)	0.806	1.32 (0.93-1.86)	0.118
> 545 days	54/585 (9.2)	212/2,340 (9.1)	1.02 (0.74-1.39)	0.912	1.22 (0.95-1.58)	0.122
Urban residents (n=2,080)						
< 180 days	358/416 (86.1)	1,428/1,664 (85.8)	1		1	
180 to 545 days	20/416 (4.8)	85/1,664 (5.1)	0.94 (0.57-1.55)	0.804	1.18 (0.76-1.83)	0.472
> 545 days	38/416 (9.1)	151/1,664 (9.1)	1.00 (0.69-1.46)	0.984	1.21 (0.89-1.66)	0.226
Rural residents (n=3,430)						
< 180 days	610/686 (88.9)	2,392/2,744 (87.2)	1		1	
180 to 545 days	33/686 (4.8)	140/2,744 (5.1)	0.92 (0.63-1.36)	0.692	1.61 (1.16-2.25)	0.005*
> 545 days	43/686 (6.3)	212/2,744 (7.7)	0.80 (0.57-1.12)	0.187	1.10 (0.84-1.46)	0.490
Underweight (n=211)						
< 180 days	78/78 (100.0)	124/133 (93.2)	1		1	
180 to 545 days	0/78 (0.0)	3/133 (2.3)	N/A		N/A	
> 545 days	0/78 (0.0)	6/133 (4.5)	N/A		N/A	
Normal weight (n=2,138)						

< 180 days	473/521 (90.8)	1,456/1,617 (90.0)	1		1	
180 to 545 days	21/521 (4.0)	62/1,617 (3.8)	1.04 (0.63-1.73)	0.872	1.45 (0.91-2.30)	0.116
> 545 days	27/521 (5.2)	99/1,617 (6.1)	0.84 (0.54-1.30)	0.434	0.95 (0.65-1.38)	0.779
Overweight (n=1,503)						
< 180 days	223/266 (83.8)	1,057/1,237 (85.4)	1		1	
180 to 545 days	20/266 (7.5)	70/1,237 (5.7)	1.35 (0.81-2.27)	0.250	2.09 (1.32-3.31)	0.002*
> 545 days	23/266 (8.6)	110/1,237 (8.9)	0.99 (0.62-1.59)	0.970	0.97 (0.66-1.41)	0.856
Obese (n=1,658)						
< 180 days	194/237 (81.9)	1,183/1,421 (83.3)	1		1	
180 to 545 days	12/237 (5.1)	90/1,421 (6.3)	0.81 (0.44-1.51)	0.514	1.01 (0.63-1.61)	0.981
> 545 days	31/237 (13.1)	148/1,421 (10.4)	1.28 (0.84-1.94)	0.249	1.77 (1.26-2.49)	0.001*
Non-smoker (n=2,622)						
< 180 days	345/403 (85.6)	1,929/2,219 (86.9)	1		1	
180 to 545 days	23/403 (5.7)	119/2,219 (5.4)	1.08 (0.68-1.71)	0.742	1.46 (1.03-2.09)	0.035*
> 545 days	35/403 (8.7)	171/2,219 (7.7)	1.14 (0.78-1.68)	0.488	1.34 (0.99-1.81)	0.057
Past and current smoker (n=2,808)						
< 180 days	623/699 (89.1)	1,891/2,189 (86.4)	1		1	
180 to 545 days	30/699 (4.3)	106/2,189 (4.8)	0.86 (0.57-1.30)	0.473	1.39 (0.95-2.05)	0.093
> 545 days	46/699 (6.6)	192/2,189 (8.8)	0.73 (0.52-1.02)	0.062	1.03 (0.77-1.38)	0.825
Alcohol consumption <1 time a week (n=2,702)						

< 180 days	368/437 (84.2)	1,962/2,265 (86.6)	1		1	
180 to 545 days	26/437 (5.9)	118/2,265 (5.2)	1.17 (0.76-1.82)	0.472	1.67 (1.15-2.42)	0.007*
> 545 days	43/437 (9.8)	185/2,265 (8.2)	1.24 (0.87-1.76)	0.230	1.91 (1.40-2.60)	<.0001
Alcohol consumption ≥1 time a week (n=2,808)						
< 180 days	600/665 (90.2)	1,858/2,143 (86.7)	1		1	
180 to 545 days	27/665 (4.1)	107/2,143 (5.0)	0.78 (0.51-1.20)	0.263	1.31 (0.89-1.91)	0.168
> 545 days	38/665 (5.7)	178/2,143 (8.3)	0.66 (0.46-0.95)	0.025*	0.78 (0.58-1.05)	0.099
SBP < 140 mmHg and DBP < 90 mmHg (n=3,811)						
< 180 days	688/776 (88.7)	2,638/3,035 (86.9)	1		1	
180 to 545 days	34/776 (4.4)	148/3,035 (4.9)	0.88 (0.60-1.29)	0.515	1.21 (0.88-1.65)	0.244
> 545 days	54/776 (7.0)	249/3,035 (8.2)	0.83 (0.61-1.13)	0.237	1.02 (0.79-1.31)	0.897
SBP ≥ 140 mmHg or DBP ≥ 90 mmHg (n=1,699)						
< 180 days	280/326 (85.9)	1,182/1,373 (86.1)	1		1	
180 to 545 days	19/326 (5.8)	77/1,373 (5.6)	1.04 (0.62-1.75)	0.876	1.99 (1.22-3.26)	0.006*
> 545 days	27/326 (8.3)	114/1,373 (8.3)	1.00 (0.64-1.55)	0.999	1.57 (1.07-2.30)	0.020*
Fasting blood glucose < 100 mg/dL (n=2,944)						
< 180 days	526/585 (89.9)	2,118/2,359 (89.8)	1		1	
180 to 545 days	25/585 (4.3)	91/2,359 (3.9)	1.11 (0.70-1.74)	0.662	1.72 (1.15-2.58)	0.008*
> 545 days	34/585 (5.8)	150/2,359 (6.4)	0.91 (0.62-1.34)	0.641	1.21 (0.88-1.65)	0.245
Fasting blood glucose ≥ 100 mg/dL (n=2,566)						

< 180 days	442/517 (85.5)	1,702/2,049 (83.1)	1		1	
180 to 545 days	28/517 (5.4)	134/2,049 (6.5)	0.80 (0.53-1.23)	0.311	1.22 (0.86-1.74)	0.272
> 545 days	47/517 (9.1)	213/2,049 (10.4)	0.85 (0.61-1.19)	0.337	1.10 (0.83-1.45)	0.498
Total cholesterol < 200 mg/dL (n=3,415)						
< 180 days	630/719 (87.6)	2,283/2,696 (84.7)	1		1	
180 to 545 days	28/719 (3.9)	119/2,696 (4.4)	0.85 (0.56-1.30)	0.458	1.47 (1.02-2.12)	0.037*
> 545 days	61/719 (8.5)	294/2,696 (10.9)	0.75 (0.56-1.00)	0.054	0.91 (0.72-1.17)	0.470
Total cholesterol ≥ 200 mg/dL (n=2,095)						
< 180 days	338/383 (88.3)	1,537/1,712 (89.8)	1		1	
180 to 545 days	25/383 (6.5)	106/1,712 (6.2)	1.07 (0.68-1.68)	0.761	1.37 (0.93-2.01)	0.107
> 545 days	20/383 (5.2)	69/1,712 (4.0)	1.32 (0.79-2.20)	0.290	2.55 (1.64-3.97)	<.0001
CCI scores = 0 (n=2,351)						
< 180 days	23/24 (95.8)	2,053/2,327 (88.2)	1		1	
180 to 545 days	1/24 (4.2)	115/2,327 (4.9)	0.78 (0.10-5.80)	0.805	0.63 (0.34-1.18)	0.147
> 545 days	0/24 (0.0)	159/2,327 (6.8)	N/A		N/A	
CCI scores = 1 (n=909)						
< 180 days	20/25 (80.0)	751/884 (85.0)	1		1	
180 to 545 days	2/25 (8.0)	45/884 (5.1)	1.67 (0.38-7.36)	0.499	2.52 (1.18-5.38)	0.017*
> 545 days	3/25 (12.0)	88/884 (10.0)	1.28 (0.37-4.39)	0.695	1.53 (0.80-2.89)	0.197
CCI scores ≥ 2 (n=2,250)						

< 180 days	925/1,053 (87.8)	1,016/1,197 (84.9)	1		1	
180 to 545 days	50/1,053 (4.7)	65/1,197 (5.4)	0.84 (0.58-1.23)	0.384	1.54 (0.97-2.42)	0.065
> 545 days	78/1,053 (7.4)	116/1,197 (9.7)	0.74 (0.55-1.00)	0.048*	1.19 (0.85-1.67)	0.306
Non dyslipidemia history (n=2,866)						
< 180 days	678/695 (97.6)	2,113/2,171 (97.3)	1		1	
180 to 545 days	6/695 (0.9)	20/2,171 (0.9)	0.93 (0.37-2.34)	0.886	0.80 (0.38-1.67)	0.551
> 545 days	11/695 (1.6)	38/2,171 (1.8)	0.90 (0.46-1.77)	0.766	0.53 (0.31-0.91)	0.022*
Dyslipidemia history (n=2,644)						
< 180 days	290/407 (71.3)	1,707/2,237 (76.3)	1		1	
180 to 545 days	47/407 (11.5)	205/2,237 (9.2)	1.35 (0.96-1.90)	0.084	1.55 (1.19-2.04)	0.001*
> 545 days	70/407 (17.2)	325/2,237 (14.5)	1.27 (0.95-1.69)	0.105	1.27 (1.02-1.59)	0.030*

Abbreviations: CCI, Charlson Comorbidity Index; SBP, Systolic blood pressure; DBP, Diastolic blood pressure;

* Significance at $P < 0.05$

† Adjusted for age, sex, income, region of residence, SBP, DBP, fasting blood glucose, total cholesterol, obesity, smoking, alcohol consumption, dyslipidemia history, and CCI scores.
