# Supplementary material for: Relation of Statin Use with Esophageal Cancer
Source: Pharmaceuticals (Basel). 2023 Jun 19;16(6):900. doi: 10.3390/ph16060900 (PMC10305244; doi:10.3390/ph16060900)
Supplement: Supplementary file 1 [file pharmaceuticals-16-00900-s001.zip › table S4.pdf]

S4 table Crude and overlap propensity score weighted odd ratios of dates of statin prescription for mortality in esophageal cancer participants.

Characteristics	Dead participants	Survived participants	Odd ratios for mortality (95% confidence interval)			
	(exposure/total, %)	(exposure/total, %)	Crude	P-value	Overlap weighted model †	P-value
Age < 65 years old (n=392)						
< 180 days	176/199 (88.4)	166/193 (86.0)	1		1	
180 to 545 days	11/199 (5.5)	14/193 (7.3)	0.74 (0.33-1.68)	0.473	1.85 (0.75-4.55)	0.178
> 545 days	12/199 (6.0)	13/193 (6.7)	0.87 (0.39-1.96)	0.738	2.00 (0.82-4.91)	0.129
Age ≥ 65 years old (n=710)						
< 180 days	398/469 (84.9)	174/241 (72.2)	1		1	
180 to 545 days	22/469 (4.7)	18/241 (7.5)	0.53 (0.28-1.02)	0.058	0.78 (0.38-1.60)	0.501
> 545 days	49/469 (10.4)	49/241 (20.3)	0.44 (0.28-0.67)	<0.001*	0.89 (0.55-1.45)	0.638
Male (n=1,021)						
< 180 days	546/631 (86.5)	306/390 (78.5)	1		1	
180 to 545 days	29/631 (4.6)	26/390 (6.7)	0.63 (0.36-1.08)	0.093	1.11 (0.61-1.99)	0.738
> 545 days	56/631 (8.9)	58/390 (14.9)	0.54 (0.37-0.80)	0.002*	1.12 (0.73-1.71)	0.617
Female (n=81)						
< 180 days	28/37 (75.7)	34/44 (77.3)	1		1	
180 to 545 days	4/37 (10.8)	6/44 (13.6)	0.81 (0.21-3.16)	0.761	1.06 (0.14-7.82)	0.951
> 545 days	5/37 (13.5)	4/44 (9.1)	1.52 (0.37-6.20)	0.561	0.51 (0.07-3.90)	0.520
Low income groups (n=517)						

< 180 days	283/327 (86.5)	157/190 (82.6)	1		1	
180 to 545 days	20/327 (6.1)	10/190 (5.3)	1.11 (0.51-2.43)	0.795	2.31 (0.96-5.56)	0.061
> 545 days	24/327 (7.3)	23/190 (12.1)	0.58 (0.32-1.06)	0.076	1.24 (0.64-2.43)	0.524
High income groups (n=585)						
< 180 days	291/341 (85.3)	183/244 (75.0)	1		1	
180 to 545 days	13/341 (3.8)	22/244 (9.0)	0.37 (0.18-0.76)	0.006*	0.54 (0.26-1.15)	0.110
> 545 days	37/341 (10.9)	39/244 (16.0)	0.60 (0.37-0.97)	0.037*	0.79 (0.46-1.39)	0.419
Urban residents (n=416)						
< 180 days	194/235 (82.6)	138/181 (76.2)	1		1	
180 to 545 days	14/235 (6.0)	11/181 (6.1)	0.91 (0.40-2.05)	0.812	1.03 (0.42-2.52)	0.946
> 545 days	27/235 (11.5)	32/181 (17.7)	0.60 (0.34-1.05)	0.072	0.87 (0.46-1.63)	0.668
Rural residents (n=686)						
< 180 days	380/433 (87.8)	202/253 (79.8)	1		1	
180 to 545 days	19/433 (4.4)	21/253 (8.3)	0.48 (0.25-0.92)	0.026*	0.90 (0.45-1.82)	0.768
> 545 days	34/433 (7.9)	30/253 (11.9)	0.60 (0.36-1.01)	0.056	1.23 (0.69-2.18)	0.484
Underweight (n=78)						
< 180 days	60/61 (98.4)	17/17 (100.0)	1		1	
180 to 545 days	1/61 (1.6)	0/17 (0.0)	N/A		N/A	
> 545 days	0/61 (0.0)	0/17 (0.0)	N/A		N/A	
Normal weight (n=521)						

< 180 days	313/352 (88.9)	138/169 (81.7)	1		1	
180 to 545 days	16/352 (4.5)	13/169 (7.7)	0.54 (0.25-1.16)	0.114	1.33 (0.58-3.05)	0.504
> 545 days	23/352 (6.5)	18/169 (10.7)	0.56 (0.29-1.08)	0.083	1.18 (0.59-2.33)	0.643
Overweight (n=266)						
< 180 days	117/144 (81.3)	92/122 (75.4)	1		1	
180 to 545 days	10/144 (6.9)	11/122 (9.0)	0.71 (0.29-1.76)	0.464	1.02 (0.38-2.72)	0.973
> 545 days	17/144 (11.8)	19/122 (15.6)	0.70 (0.35-1.43)	0.331	1.63 (0.69-3.84)	0.267
Obese (n=237)						
< 180 days	84/111 (75.7)	93/126 (73.8)	1		1	
180 to 545 days	6/111 (5.4)	8/126 (6.3)	0.83 (0.28-2.49)	0.740	0.98 (0.26-3.72)	0.982
> 545 days	21/111 (18.9)	25/126 (19.8)	0.93 (0.49-1.78)	0.827	0.63 (0.29-1.39)	0.253
Non-smoker (n=403)						
< 180 days	195/241 (80.9)	132/162 (81.5)	1		1	
180 to 545 days	16/241 (6.6)	12/162 (7.4)	0.90 (0.41-1.97)	0.797	1.36 (0.58-3.16)	0.479
> 545 days	30/241 (12.4)	18/162 (11.1)	1.13 (0.60-2.11)	0.705	1.45 (0.76-2.79)	0.264
Past and current smoker (n=699)						
< 180 days	379/427 (88.8)	208/272 (76.5)	1		1	
180 to 545 days	17/427 (4.0)	20/272 (7.4)	0.47 (0.24-0.91)	0.025*	0.76 (0.36-1.59)	0.471
> 545 days	31/427 (7.3)	44/272 (16.2)	0.39 (0.24-0.63)	<0.001*	0.87 (0.50-1.51)	0.622
Alcohol consumption <1 time a week (n=437)						

< 180 days	223/275 (81.1)	123/162 (75.9)	1		1	
180 to 545 days	16/275 (5.8)	13/162 (8.0)	0.68 (0.32-1.46)	0.321	0.87 (0.38-2.01)	0.752
> 545 days	36/275 (13.1)	26/162 (16.0)	0.76 (0.44-1.32)	0.337	1.11 (0.61-2.05)	0.727
Alcohol consumption ≥1 time a week (n=665)						
< 180 days	351/393 (89.3)	217/272 (79.8)	1		1	
180 to 545 days	17/393 (4.3)	19/272 (7.0)	0.55 (0.28-1.09)	0.086	1.11 (0.53-2.31)	0.781
> 545 days	25/393 (6.4)	36/272 (13.2)	0.43 (0.25-0.73)	0.002*	0.92 (0.52-1.65)	0.789
SBP < 140 mmHg and DBP < 90 mmHg (n=776)						
< 180 days	384/445 (86.3)	258/331 (77.9)	1		1	
180 to 545 days	21/445 (4.7)	25/331 (7.6)	0.56 (0.31-1.03)	0.062	1.13 (0.58-2.22)	0.715
> 545 days	40/445 (9.0)	48/331 (14.5)	0.56 (0.36-0.88)	0.011*	1.35 (0.82-2.24)	0.240
SBP ≥ 140 mmHg or DBP ≥ 90 mmHg (n=326)						
< 180 days	190/223 (85.2)	82/103 (79.6)	1		1	
180 to 545 days	12/223 (5.4)	7/103 (6.8)	0.74 (0.28-1.95)	0.542	0.63 (0.23-1.70)	0.360
> 545 days	21/223 (9.4)	14/103 (13.6)	0.65 (0.31-1.34)	0.239	0.68 (0.30-1.53)	0.352
Fasting blood glucose < 100 mg/dL (n=585)						
< 180 days	322/361 (89.2)	182/224 (81.3)	1		1	
180 to 545 days	14/361 (3.9)	18/224 (8.0)	0.44 (0.21-0.90)	0.026*	0.67 (0.31-1.46)	0.315
> 545 days	25/361 (6.9)	24/224 (10.7)	0.59 (0.33-1.06)	0.078	1.11 (0.58-2.13)	0.757
Fasting blood glucose ≥ 100 mg/dL (n=517)						

< 180 days	252/307 (82.1)	158/210 (75.2)	1		1	
180 to 545 days	19/307 (6.2)	14/210 (6.7)	0.85 (0.41-1.75)	0.660	1.63 (0.72-3.67)	0.240
> 545 days	36/307 (11.7)	38/210 (18.1)	0.59 (0.36-0.98)	0.040*	1.11 (0.63-1.96)	0.712
Total cholesterol < 200 mg/dL (n=719)						
< 180 days	377/443 (85.1)	210/276 (76.1)	1		1	
180 to 545 days	20/443 (4.5)	16/276 (5.8)	0.70 (0.35-1.37)	0.296	1.87 (0.91-3.83)	0.086
> 545 days	46/443 (10.4)	50/276 (18.1)	0.51 (0.33-0.79)	0.003*	1.04 (0.64-1.68)	0.868
Total cholesterol ≥ 200 mg/dL (n=383)						
< 180 days	197/225 (87.6)	130/158 (82.3)	1		1	
180 to 545 days	13/225 (5.8)	16/158 (10.1)	0.54 (0.25-1.15)	0.110	0.36 (0.14-0.95)	0.038*
> 545 days	15/225 (6.7)	12/158 (7.6)	0.82 (0.37-1.82)	0.633	1.36 (0.53-3.49)	0.521
CCI scores = 0 (n=24)						
< 180 days	9/9 (100.0)	13/15 (86.7)	1		1	
180 to 545 days	0/9 (0.0)	1/15 (6.7)	N/A		N/A	
> 545 days	0/9 (0.0)	1/15 (6.7)	N/A		N/A	
CCI scores = 1 (n=25)						
< 180 days	8/8 (100.0)	10/17 (58.8)	1		1	
180 to 545 days	0/8 (0.0)	4/17 (23.5)	N/A		N/A	
> 545 days	0/8 (0.0)	3/17 (17.6)	N/A		N/A	
CCI scores ≥ 2 (n=1,053)						

< 180 days	557/651 (85.6)	317/402 (78.9)	1		1	
180 to 545 days	33/651 (5.1)	27/402 (6.7)	0.70 (0.41-1.18)	0.177	1.09 (0.63-1.89)	0.766
> 545 days	61/651 (9.4)	58/402 (14.4)	0.60 (0.41-0.88)	0.009*	1.09 (0.71-1.66)	0.696
Non dyslipidemia history (n=695)						
< 180 days	469/485 (96.7)	202/210 (96.2)	1		1	
180 to 545 days	5/485 (1.0)	4/210 (1.9)	0.54 (0.14-2.03)	0.360	0.44 (0.10-1.97)	0.282
> 545 days	11/485 (2.3)	4/210 (1.9)	1.18 (0.37-3.76)	0.774	1.31 (0.46-3.74)	0.612
Dyslipidemia history (n=407)						
< 180 days	105/183 (57.4)	138/224 (61.6)	1		1	
180 to 545 days	28/183 (15.3)	28/224 (12.5)	1.31 (0.73-2.35)	0.358	1.16 (0.62-2.17)	0.647
> 545 days	50/183 (27.3)	58/224 (25.9)	1.13 (0.72-1.79)	0.591	0.93 (0.56-1.53)	0.770
No treatment (n=374)						
< 180 days	176/204 (86.3)	127/170 (74.7)	1		1	
180 to 545 days	11/204 (5.4)	15/170 (8.8)	0.53 (0.24-1.19)	0.124	1.08 (0.45-2.57)	0.863
> 545 days	17/204 (8.3)	28/170 (16.5)	0.44 (0.23-0.83)	0.012*	0.90 (0.42-1.94)	0.788
Only surgery (n=163)						
< 180 days	49/59 (83.1)	84/104 (80.8)	1		1	
180 to 545 days	0/59 (0.0)	7/104 (6.7)	N/A		N/A	
> 545 days	10/59 (16.9)	13/104 (12.5)	1.32 (0.54-3.23)	0.545	1.96 (0.59-6.46)	0.271

Surgery or radiotherapy or chemotherapy (n=565)						
< 180 days	349/405 (86.2)	129/160 (80.6)	1		1	
180 to 545 days	22/405 (5.4)	10/160 (6.3)	0.81 (0.37-1.76)	0.601	1.53 (0.68-3.46)	0.308
> 545 days	34/405 (8.4)	21/160 (13.1)	0.60 (0.33-1.07)	0.083	0.98 (0.53-1.83)	0.960

Abbreviations: CCI, Charlson Comorbidity Index; SBP, Systolic blood pressure; DBP, Diastolic blood pressure;

* Significance at P < 0.05

† Adjusted for age, sex, income, region of residence, SBP, DBP, fasting blood glucose, total cholesterol, obesity, smoking, alcohol consumption, dyslipidemia history, CCI scores and treatment type.
