# Supplementary material for: Relation of Statin Use with Esophageal Cancer
Source: Pharmaceuticals (Basel). 2023 Jun 19;16(6):900. doi: 10.3390/ph16060900 (PMC10305244; doi:10.3390/ph16060900)
Supplement: Supplementary file 1 [file pharmaceuticals-16-00900-s001.zip › table S5.pdf]

S5 table Crude and overlap propensity score weighted odd ratios of dates of hydrophilic prescription for mortality in esophageal cancer participants.

Characteristics	Dead participants	Survived participants	Odd ratios for mortality (95% confidence interval)			
	(exposure/total, %)	(exposure/total, %)	Crude	P-value	Overlap weighted model †	P-value
Age < 65 years old (n=392)						
< 180 days	176/199 (88.4)	166/193 (86.0)	1		1	
180 to 545 days	11/199 (5.5)	14/193 (7.3)	0.47 (0.09-2.62)	0.392	0.82 (0.09-7.32)	0.859
> 545 days	12/199 (6.0)	13/193 (6.7)	0.63 (0.18-2.28)	0.483	0.75 (0.22-2.58)	0.651
Age ≥ 65 years old (n=710)						
< 180 days	398/469 (84.9)	174/241 (72.2)	1		1	
180 to 545 days	22/469 (4.7)	18/241 (7.5)	0.96 (0.32-2.84)	0.938	1.69 (0.49-5.82)	0.404
> 545 days	49/469 (10.4)	49/241 (20.3)	0.19 (0.08-0.44)	<0.001*	0.56 (0.25-1.24)	0.153
Male (n=1,021)						
< 180 days	546/631 (86.5)	306/390 (78.5)	1		1	
180 to 545 days	29/631 (4.6)	26/390 (6.7)	1.08 (0.40-2.95)	0.879	2.19 (0.64-7.49)	0.214
> 545 days	56/631 (8.9)	58/390 (14.9)	0.26 (0.13-0.53)	<0.001*	0.72 (0.37-1.40)	0.334
Female (n=81)						
< 180 days	28/37 (75.7)	34/44 (77.3)	1		1	
180 to 545 days	4/37 (10.8)	6/44 (13.6)	0.38 (0.04-3.83)	0.413	1.18 (0.07-19.0)	0.905
> 545 days	5/37 (13.5)	4/44 (9.1)	1.14 (0.07-19.0)	0.926	0.04 (0.00-29.2)	0.343
Low income groups (n=517)						

< 180 days	283/327 (86.5)	157/190 (82.6)	1		1	
180 to 545 days	20/327 (6.1)	10/190 (5.3)	0.98 (0.28-3.41)	0.980	2.27 (0.49-10.5)	0.295
> 545 days	24/327 (7.3)	23/190 (12.1)	0.39 (0.15-1.05)	0.063	1.65 (0.63-4.34)	0.308
High income groups (n=585)						
< 180 days	291/341 (85.3)	183/244 (75.0)	1		1	
180 to 545 days	13/341 (3.8)	22/244 (9.0)	0.67 (0.19-2.35)	0.536	0.96 (0.21-4.45)	0.963
> 545 days	37/341 (10.9)	39/244 (16.0)	0.21 (0.08-0.58)	0.003*	0.32 (0.12-0.85)	0.023*
Urban residents (n=416)						
< 180 days	194/235 (82.6)	138/181 (76.2)	1		1	
180 to 545 days	14/235 (6.0)	11/181 (6.1)	0.96 (0.21-4.33)	0.953	1.07 (0.15-7.77)	0.947
> 545 days	27/235 (11.5)	32/181 (17.7)	0.22 (0.08-0.62)	0.004*	0.39 (0.15-1.01)	0.052
Rural residents (n=686)						
< 180 days	380/433 (87.8)	202/253 (79.8)	1		1	
180 to 545 days	19/433 (4.4)	21/253 (8.3)	0.76 (0.26-2.20)	0.609	1.92 (0.54-6.79)	0.310
> 545 days	34/433 (7.9)	30/253 (11.9)	0.40 (0.15-1.06)	0.064	1.00 (0.39-2.59)	0.998
Underweight (n=78)						
< 180 days	60/61 (98.4)	17/17 (100.0)	1		1	
180 to 545 days	1/61 (1.6)	0/17 (0.0)	N/A		N/A	
> 545 days	0/61 (0.0)	0/17 (0.0)	N/A		N/A	
Normal weight (n=521)						

< 180 days	313/352 (88.9)	138/169 (81.7)	1		1	
180 to 545 days	16/352 (4.5)	13/169 (7.7)	0.55 (0.17-1.83)	0.328	1.17 (0.31-4.51)	0.817
> 545 days	23/352 (6.5)	18/169 (10.7)	0.29 (0.09-0.89)	0.030*	0.59 (0.20-1.77)	0.348
Overweight (n=266)						
< 180 days	117/144 (81.3)	92/122 (75.4)	1		1	
180 to 545 days	10/144 (6.9)	11/122 (9.0)	3.25 (0.36-29.5)	0.295	35.7 (0.14-9399)	0.208
> 545 days	17/144 (11.8)	19/122 (15.6)	0.18 (0.04-0.85)	0.031*	0.52 (0.13-2.09)	0.356
Obese (n=237)						
< 180 days	84/111 (75.7)	93/126 (73.8)	1		1	
180 to 545 days	6/111 (5.4)	8/126 (6.3)	0.36 (0.04-3.53)	0.382	0.06 (0.00-9.25)	0.280
> 545 days	21/111 (18.9)	25/126 (19.8)	0.60 (0.20-1.86)	0.378	0.95 (0.30-3.02)	0.930
Non-smoker (n=403)						
< 180 days	195/241 (80.9)	132/162 (81.5)	1		1	
180 to 545 days	16/241 (6.6)	12/162 (7.4)	1.06 (0.34-3.31)	0.917	2.01 (0.50-8.08)	0.323
> 545 days	30/241 (12.4)	18/162 (11.1)	0.53 (0.14-2.01)	0.351	0.74 (0.18-3.10)	0.677
Past and current smoker (n=699)						
< 180 days	379/427 (88.8)	208/272 (76.5)	1		1	
180 to 545 days	17/427 (4.0)	20/272 (7.4)	0.60 (0.15-2.40)	0.466	1.04 (0.18-6.19)	0.964
> 545 days	31/427 (7.3)	44/272 (16.2)	0.23 (0.10-0.52)	0.001*	0.64 (0.31-1.35)	0.241
Alcohol consumption <1 time a week (n=437)						

< 180 days	223/275 (81.1)	123/162 (75.9)	1		1	
180 to 545 days	16/275 (5.8)	13/162 (8.0)	1.13 (0.28-4.60)	0.861	1.18 (0.19-7.31)	0.859
> 545 days	36/275 (13.1)	26/162 (16.0)	0.34 (0.12-0.95)	0.041*	0.63 (0.23-1.72)	0.364
Alcohol consumption ≥1 time a week (n=665)						
< 180 days	351/393 (89.3)	217/272 (79.8)	1		1	
180 to 545 days	17/393 (4.3)	19/272 (7.0)	0.66 (0.21-2.06)	0.470	1.56 (0.42-5.74)	0.506
> 545 days	25/393 (6.4)	36/272 (13.2)	0.25 (0.10-0.64)	0.004*	0.59 (0.24-1.43)	0.244
SBP < 140 mmHg and DBP < 90 mmHg (n=776)						
< 180 days	384/445 (86.3)	258/331 (77.9)	1		1	
180 to 545 days	21/445 (4.7)	25/331 (7.6)	0.70 (0.27-1.77)	0.448	1.44 (0.48-4.32)	0.510
> 545 days	40/445 (9.0)	48/331 (14.5)	0.23 (0.10-0.52)	0.000*	0.51 (0.23-1.11)	0.092
SBP ≥ 140 mmHg or DBP ≥ 90 mmHg (n=326)						
< 180 days	190/223 (85.2)	82/103 (79.6)	1		1	
180 to 545 days	12/223 (5.4)	7/103 (6.8)	84E4 (0.00- I)	0.986	43E4 (0.00- I)	0.986
> 545 days	21/223 (9.4)	14/103 (13.6)	0.94 (0.17-5.19)	0.939	2.79 (0.59-13.1)	0.195
Fasting blood glucose < 100 mg/dL (n=585)						
< 180 days	322/361 (89.2)	182/224 (81.3)	1		1	
180 to 545 days	14/361 (3.9)	18/224 (8.0)	0.81 (0.18-3.63)	0.778	1.46 (0.27-7.83)	0.659
> 545 days	25/361 (6.9)	24/224 (10.7)	0.40 (0.14-1.15)	0.089	0.83 (0.31-2.24)	0.715
Fasting blood glucose ≥ 100 mg/dL (n=517)						

< 180 days	252/307 (82.1)	158/210 (75.2)	1		1	
180 to 545 days	19/307 (6.2)	14/210 (6.7)	0.85 (0.29-2.49)	0.768	1.51 (0.38-6.07)	0.562
> 545 days	36/307 (11.7)	38/210 (18.1)	0.23 (0.09-0.58)	0.002*	0.66 (0.27-1.61)	0.360
Total cholesterol < 200 mg/dL (n=719)						
< 180 days	377/443 (85.1)	210/276 (76.1)	1		1	
180 to 545 days	20/443 (4.5)	16/276 (5.8)	0.75 (0.28-2.04)	0.571	1.71 (0.53-5.48)	0.369
> 545 days	46/443 (10.4)	50/276 (18.1)	0.26 (0.12-0.57)	0.001*	0.60 (0.29-1.23)	0.161
Total cholesterol ≥ 200 mg/dL (n=383)						
< 180 days	197/225 (87.6)	130/158 (82.3)	1		1	
180 to 545 days	13/225 (5.8)	16/158 (10.1)	1.04 (0.17-6.28)	0.969	1.42 (0.10-19.9)	0.797
> 545 days	15/225 (6.7)	12/158 (7.6)	0.35 (0.06-1.91)	0.223	0.92 (0.16-5.45)	0.928
CCI scores = 0 (n=24)						
< 180 days	9/9 (100.0)	13/15 (86.7)	1		1	
180 to 545 days	0/9 (0.0)	1/15 (6.7)	N/A	0.993	N/A	0.024*
> 545 days	0/9 (0.0)	1/15 (6.7)	N/A	0.095	N/A	0.350
CCI scores = 1 (n=25)						
< 180 days	8/8 (100.0)	10/17 (58.8)	1		1	
180 to 545 days	0/8 (0.0)	4/17 (23.5)	N/A	0.280	N/A	0.110
> 545 days	0/8 (0.0)	3/17 (17.6)	N/A	0.002*	N/A	0.040*
CCI scores ≥ 2 (n=1,053)						

< 180 days	557/651 (85.6)	317/402 (78.9)	1		1	
180 to 545 days	33/651 (5.1)	27/402 (6.7)	1.18 (0.44-3.18)	0.738	2.08 (0.66-6.59)	0.212
> 545 days	61/651 (9.4)	58/402 (14.4)	0.28 (0.14-0.57)	<0.001*	0.65 (0.34-1.25)	0.199
Non dyslipidemia history (n=695)						
< 180 days	469/485 (96.7)	202/210 (96.2)	1		1	
180 to 545 days	5/485 (1.0)	4/210 (1.9)	1.29 (0.13-12.5)	0.825	1.73 (0.13-22.5)	0.677
> 545 days	11/485 (2.3)	4/210 (1.9)	0.22 (0.02-2.39)	0.211	0.12 (0.00-5.96)	0.290
Dyslipidemia history (n=407)						
< 180 days	105/183 (57.4)	138/224 (61.6)	1		1	
180 to 545 days	28/183 (15.3)	28/224 (12.5)	1.33 (0.50-3.51)	0.571	2.09 (0.60-7.29)	0.245
> 545 days	50/183 (27.3)	58/224 (25.9)	0.54 (0.26-1.14)	0.104	0.77 (0.37-1.61)	0.492
No treatment (n=374)						
< 180 days	176/204 (86.3)	127/170 (74.7)	1		1	
180 to 545 days	11/204 (5.4)	15/170 (8.8)	0.39 (0.10-1.59)	0.188	0.30 (0.03-2.57)	0.272
> 545 days	17/204 (8.3)	28/170 (16.5)	0.35 (0.12-1.04)	0.060	1.39 (0.47-4.13)	0.549
Only surgery (n=163)						
< 180 days	49/59 (83.1)	84/104 (80.8)	1		1	
180 to 545 days	0/59 (0.0)	7/104 (6.7)	0.84 (0.07-9.50)	0.889	1.69 (0.12-24.2)	0.700
> 545 days	10/59 (16.9)	13/104 (12.5)	0.28 (0.03-2.39)	0.245	0.86 (0.10-7.42)	0.887
Surgery or radiotherapy or chemotherapy (n=565)						

< 180 days	349/405 (86.2)	129/160 (80.6)	1		1	
180 to 545 days	22/405 (5.4)	10/160 (6.3)	3.07 (0.38-24.7)	0.293	4.40 (0.58-33.5)	0.152
> 545 days	34/405 (8.4)	21/160 (13.1)	0.26 (0.09-0.73)	0.011*	0.47 (0.18-1.23)	0.124

Abbreviations: CCI, Charlson Comorbidity Index; SBP, Systolic blood pressure; DBP, Diastolic blood pressure;

* Significance at $P < 0.05$

† Adjusted for age, sex, income, region of residence, SBP, DBP, fasting blood glucose, total cholesterol, obesity, smoking, alcohol consumption, dyslipidemia history, CCI scores and treatment type.
