# Supplementary material for: Relation of Statin Use with Esophageal Cancer
Source: Pharmaceuticals (Basel). 2023 Jun 19;16(6):900. doi: 10.3390/ph16060900 (PMC10305244; doi:10.3390/ph16060900)
Supplement: Supplementary file 1 [file pharmaceuticals-16-00900-s001.zip › table S6.pdf]

S6 table Crude and overlap propensity score weighted odd ratios of dates of lipophilic statin prescription for mortality in esophageal cancer participants.

Characteristics	Dead participants (exposure/total, %)	Survived participants (exposure/total, %)	Odd ratios for mortality (95% confidence interval)			
			Crude	P-value	Overlap weighted model †	P-value
Age < 65 years old (n=392)						
< 180 days	176/199 (88.4)	166/193 (86.0)	1		1	
180 to 545 days	11/199 (5.5)	14/193 (7.3)	0.97 (0.40-2.39)	0.951	2.62 (1.00-6.88)	0.051
> 545 days	12/199 (6.0)	13/193 (6.7)	1.11 (0.39-3.13)	0.842	3.84 (1.13-13.1)	0.031*
Age ≥ 65 years old (n=710)						
< 180 days	398/469 (84.9)	174/241 (72.2)	1		1	
180 to 545 days	22/469 (4.7)	18/241 (7.5)	0.65 (0.32-1.32)	0.236	0.93 (0.44-1.96)	0.848
> 545 days	49/469 (10.4)	49/241 (20.3)	0.61 (0.37-1.02)	0.061	1.05 (0.61-1.80)	0.855
Male (n=1.021)						
< 180 days	546/631 (86.5)	306/390 (78.5)	1		1	
180 to 545 days	29/631 (4.6)	26/390 (6.7)	0.78 (0.43-1.41)	0.408	1.39 (0.76-2.55)	0.284
> 545 days	56/631 (8.9)	58/390 (14.9)	0.74 (0.46-1.20)	0.222	1.29 (0.79-2.10)	0.318
Female (n=81)						
< 180 days	28/37 (75.7)	34/44 (77.3)	1		1	
180 to 545 days	4/37 (10.8)	6/44 (13.6)	0.93 (0.19-4.46)	0.923	0.98 (0.10-9.42)	0.983
> 545 days	5/37 (13.5)	4/44 (9.1)	1.64 (0.34-7.92)	0.535	0.68 (0.09-5.06)	0.709

Low income groups (n=517)						
< 180 days	283/327 (86.5)	157/190 (82.6)	1		1	
180 to 545 days	20/327 (6.1)	10/190 (5.3)	1.84 (0.72-4.70)	0.201	2.84 (1.08-7.46)	0.034*
> 545 days	24/327 (7.3)	23/190 (12.1)	0.63 (0.29-1.36)	0.239	0.82 (0.37-1.85)	0.636
High income groups (n=585)						
< 180 days	291/341 (85.3)	183/244 (75.0)	1		1	
180 to 545 days	13/341 (3.8)	22/244 (9.0)	0.38 (0.17-0.83)	0.016*	0.73 (0.34-1.55)	0.410
> 545 days	37/341 (10.9)	39/244 (16.0)	0.91 (0.52-1.61)	0.751	1.21 (0.65-2.25)	0.544
Urban residents (n=416)						
< 180 days	194/235 (82.6)	138/181 (76.2)	1		1	
180 to 545 days	14/235 (6.0)	11/181 (6.1)	0.94 (0.38-2.33)	0.900	1.21 (0.48-3.05)	0.683
> 545 days	27/235 (11.5)	32/181 (17.7)	1.06 (0.54-2.09)	0.863	1.48 (0.72-3.06)	0.285
Rural residents (n=686)						
< 180 days	380/433 (87.8)	202/253 (79.8)	1		1	
180 to 545 days	19/433 (4.4)	21/253 (8.3)	0.67 (0.33-1.35)	0.261	1.25 (0.60-2.62)	0.553
> 545 days	34/433 (7.9)	30/253 (11.9)	0.64 (0.34-1.19)	0.159	1.12 (0.58-2.14)	0.741
Underweight (n=78)						
< 180 days	60/61 (98.4)	17/17 (100.0)	1		1	
180 to 545 days	1/61 (1.6)	0/17 (0.0)	N/A		N/A	
> 545 days	0	0	N/A		N/A	

Normal weight (n=521)						
< 180 days	313/352 (88.9)	138/169 (81.7)	1		1	
180 to 545 days	16/352 (4.5)	13/169 (7.7)	0.76 (0.31-1.88)	0.555	2.02 (0.78-5.20)	0.147
> 545 days	23/352 (6.5)	18/169 (10.7)	0.80 (0.36-1.78)	0.581	1.57 (0.72-3.41)	0.256
Overweight (n=266)						
< 180 days	117/144 (81.3)	92/122 (75.4)	1		1	
180 to 545 days	10/144 (6.9)	11/122 (9.0)	0.68 (0.27-1.70)	0.406	1.16 (0.45-3.02)	0.756
> 545 days	17/144 (11.8)	19/122 (15.6)	1.08 (0.45-2.56)	0.868	2.26 (0.80-6.41)	0.126
Obese (n=237)						
< 180 days	84/111 (75.7)	93/126 (73.8)	1		1	
180 to 545 days	6/111 (5.4)	8/126 (6.3)	1.65 (0.51-5.39)	0.405	1.71 (0.40-7.23)	0.469
> 545 days	21/111 (18.9)	25/126 (19.8)	1.11 (0.52-2.36)	0.795	0.59 (0.24-1.42)	0.238
Non-smoker (n=403)						
< 180 days	195/241 (80.9)	132/162 (81.5)	1		1	
180 to 545 days	16/241 (6.6)	12/162 (7.4)	1.30 (0.54-3.14)	0.566	1.93 (0.77-4.86)	0.160
> 545 days	30/241 (12.4)	18/162 (11.1)	1.17 (0.57-2.40)	0.669	1.40 (0.69-2.82)	0.352
Past and current smoker (n=699)						
< 180 days	379/427 (88.8)	208/272 (76.5)	1		1	
180 to 545 days	17/427 (4.0)	20/272 (7.4)	0.52 (0.25-1.09)	0.084	0.91 (0.42-1.95)	0.805
> 545 days	31/427 (7.3)	44/272 (16.2)	0.60 (0.33-1.09)	0.093	1.23 (0.63-2.39)	0.539

Alcohol consumption <1 time a week (n=437)						
< 180 days	223/275 (81.1)	123/162 (75.9)	1		1	
180 to 545 days	16/275 (5.8)	13/162 (8.0)	0.79 (0.35-1.77)	0.567	1.06 (0.44-2.58)	0.891
> 545 days	36/275 (13.1)	26/162 (16.0)	0.98 (0.51-1.88)	0.946	1.37 (0.70-2.67)	0.361
Alcohol consumption ≥1 time a week (n=665)						
< 180 days	351/393 (89.3)	217/272 (79.8)	1		1	
180 to 545 days	17/393 (4.3)	19/272 (7.0)	0.71 (0.33-1.54)	0.391	1.54 (0.72-3.31)	0.263
> 545 days	25/393 (6.4)	36/272 (13.2)	0.60 (0.31-1.15)	0.123	1.11 (0.55-2.23)	0.771
SBP < 140 mmHg and DBP < 90 mmHg (n=776)						
< 180 days	384/445 (86.3)	258/331 (77.9)	1		1	
180 to 545 days	21/445 (4.7)	25/331 (7.6)	0.73 (0.37-1.45)	0.368	1.71 (0.82-3.54)	0.150
> 545 days	40/445 (9.0)	48/331 (14.5)	0.91 (0.52-1.59)	0.743	2.13 (1.16-3.91)	0.015*
SBP ≥ 140 mmHg or DBP ≥ 90 mmHg (n=326)						
< 180 days	190/223 (85.2)	82/103 (79.6)	1		1	
180 to 545 days	12/223 (5.4)	7/103 (6.8)	0.73 (0.28-1.93)	0.532	0.58 (0.21-1.57)	0.279
> 545 days	21/223 (9.4)	14/103 (13.6)	0.54 (0.24-1.19)	0.127	0.44 (0.18-1.06)	0.066
Fasting blood glucose < 100 mg/dL (n=585)						
< 180 days	322/361 (89.2)	182/224 (81.3)	1		1	
180 to 545 days	14/361 (3.9)	18/224 (8.0)	0.46 (0.21-1.04)	0.062	0.74 (0.32-1.70)	0.482
> 545 days	25/361 (6.9)	24/224 (10.7)	0.75 (0.37-1.50)	0.412	1.35 (0.64-2.83)	0.432

Fasting blood glucose ≥ 100 mg/dL (n=517)						
< 180 days	252/307 (82.1)	158/210 (75.2)	1		1	
180 to 545 days	19/307 (6.2)	14/210 (6.7)	1.23 (0.55-2.72)	0.617	2.23 (0.95-5.22)	0.066
> 545 days	36/307 (11.7)	38/210 (18.1)	0.84 (0.46-1.54)	0.580	1.23 (0.65-2.33)	0.531
Total cholesterol < 200 mg/dL (n=719)						
< 180 days	377/443 (85.1)	210/276 (76.1)	1		1	
180 to 545 days	20/443 (4.5)	16/276 (5.8)	0.93 (0.43-2.02)	0.859	2.71 (1.26-5.87)	0.011*
> 545 days	46/443 (10.4)	50/276 (18.1)	0.71 (0.42-1.21)	0.206	1.22 (0.72-2.10)	0.460
Total cholesterol ≥ 200 mg/dL (n=383)						
< 180 days	197/225 (87.6)	130/158 (82.3)	1		1	
180 to 545 days	13/225 (5.8)	16/158 (10.1)	0.63 (0.28-1.42)	0.265	0.38 (0.14-1.07)	0.066
> 545 days	15/225 (6.7)	12/158 (7.6)	1.02 (0.41-2.57)	0.962	1.59 (0.54-4.71)	0.402
CCI scores = 0 (n=24)						
< 180 days	9/9 (100.0)	13/15 (86.7)	1		1	
180 to 545 days	0/9 (0.0)	1/15 (6.7)	N/A		N/A	
> 545 days	0/9 (0.0)	1/15 (6.7)	N/A		N/A	
CCI scores = 1 (n=25)						
< 180 days	8/8 (100.0)	10/17 (58.8)	1		1	
180 to 545 days	0/8 (0.0)	4/17 (23.5)	N/A		N/A	
> 545 days	0/8 (0.0)	3/17 (17.6)	N/A		N/A	

CCI scores ≥ 2 (n=1,053)						
< 180 days	557/651 (85.6)	317/402 (78.9)	1		1	
180 to 545 days	33/651 (5.1)	27/402 (6.7)	0.83 (0.47-1.48)	0.535	1.33 (0.75-2.36)	0.333
> 545 days	61/651 (9.4)	58/402 (14.4)	0.82 (0.51-1.31)	0.413	1.31 (0.81-2.11)	0.276
Non dyslipidemia history (n=695)						
< 180 days	469/485 (96.7)	202/210 (96.2)	1		1	
180 to 545 days	5/485 (1.0)	4/210 (1.9)	0.43 (0.09-2.17)	0.308	0.27 (0.04-1.74)	0.167
> 545 days	11/485 (2.3)	4/210 (1.9)	1.95 (0.42-9.11)	0.396	1.99 (0.60-6.60)	0.263
Dyslipidemia history (n=407)						
< 180 days	105/183 (57.4)	138/224 (61.6)	1		1	
180 to 545 days	28/183 (15.3)	28/224 (12.5)	1.73 (0.93-3.22)	0.084	1.47 (0.77-2.82)	0.239
> 545 days	50/183 (27.3)	58/224 (25.9)	1.48 (0.88-2.50)	0.143	0.97 (0.55-1.70)	0.908
No treatment (n=374)						
< 180 days	176/204 (86.3)	127/170 (74.7)	1		1	
180 to 545 days	11/204 (5.4)	15/170 (8.8)	0.70 (0.28-1.77)	0.450	1.57 (0.61-4.05)	0.355
> 545 days	17/204 (8.3)	28/170 (16.5)	0.50 (0.23-1.11)	0.088	0.68 (0.27-1.73)	0.416
Only surgery (n=163)						
< 180 days	49/59 (83.1)	84/104 (80.8)	1		1	
180 to 545 days	0/59 (0.0)	7/104 (6.7)	0.37 (0.04-3.24)	0.368	0.34 (0.04-2.63)	0.300

> 545 days	10/59 (16.9)	13/104 (12.5)	2.10 (0.72-6.14)	0.174	2.12 (0.55-8.19)	0.277
Surgery or radiotherapy or chemotherapy (n=565)						
< 180 days	349/405 (86.2)	129/160 (80.6)	1		1	
180 to 545 days	22/405 (5.4)	10/160 (6.3)	0.82 (0.36-1.84)	0.624	1.53 (0.66-3.57)	0.325
> 545 days	34/405 (8.4)	21/160 (13.1)	0.84 (0.41-1.70)	0.623	1.38 (0.69-2.77)	0.361

Abbreviations: CCI, Charlson Comorbidity Index; SBP, Systolic blood pressure; DBP, Diastolic blood pressure;

* Significance at P < 0.05

† Adjusted for age, sex, income, region of residence, SBP, DBP, fasting blood glucose, total cholesterol, obesity, smoking, alcohol consumption, dyslipidemia history, CCI scores and treatment type.
