# Supplementary figures and images for: Relation of Statin Use with Esophageal Cancer
Source: Pharmaceuticals (Basel). 2023 Jun 19;16(6):900. doi: 10.3390/ph16060900 (PMC10305244; doi:10.3390/ph16060900)

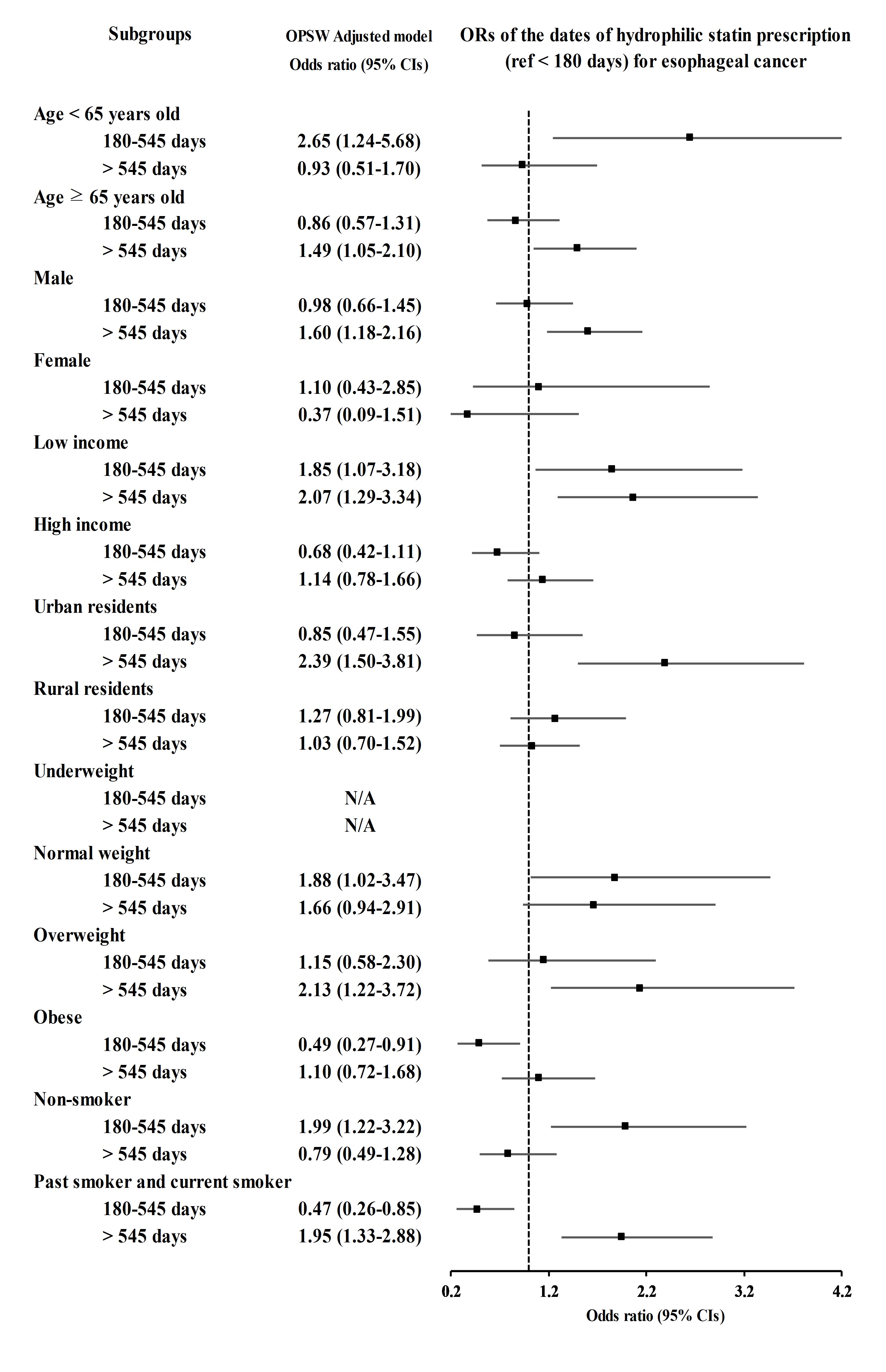

Supplement: Supplementary file 1 [file pharmaceuticals-16-00900-s001.zip › Fig S2A.JPG]

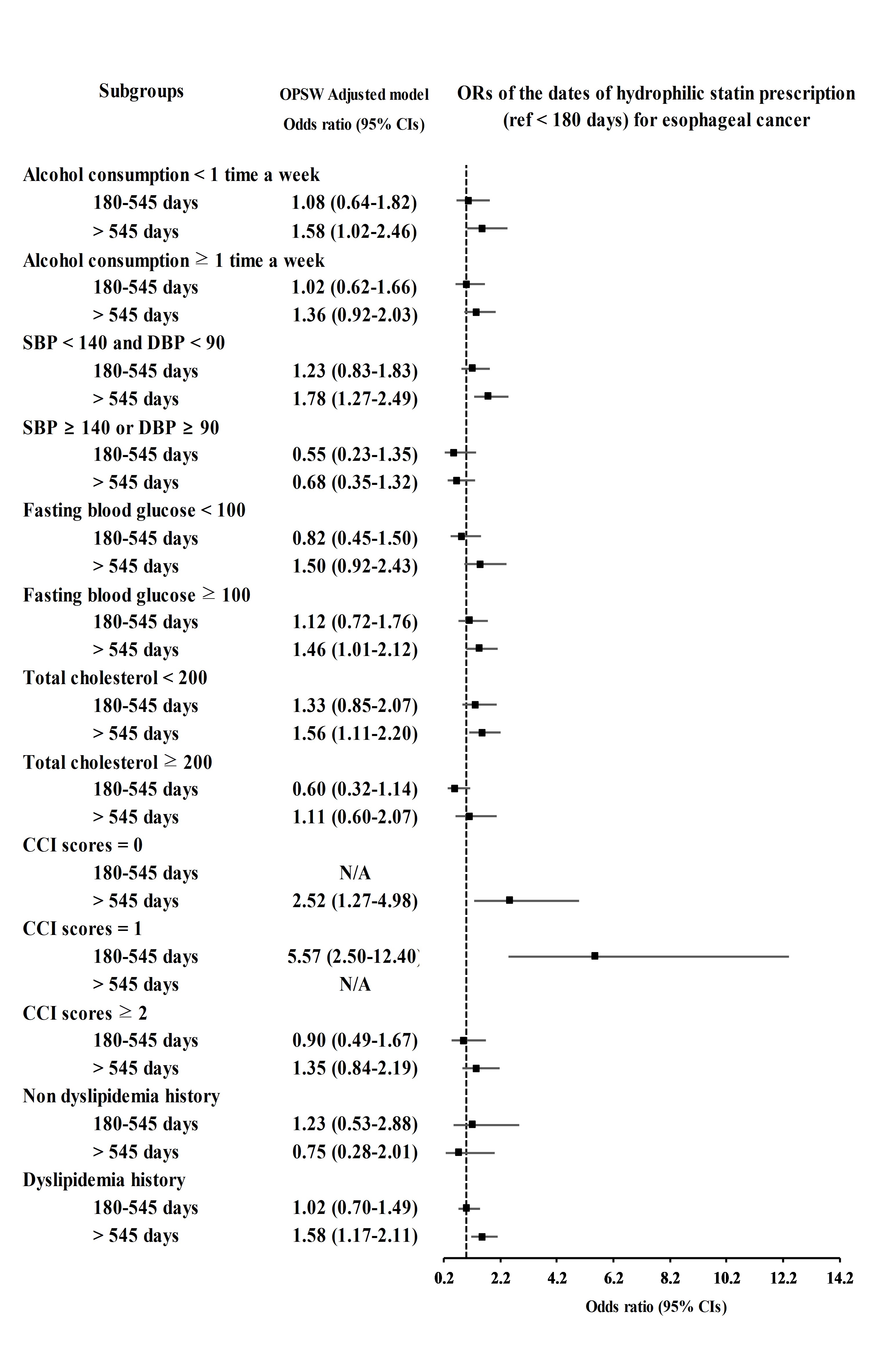

Supplement: Supplementary file 1 [file pharmaceuticals-16-00900-s001.zip › Fig S2B.JPG]

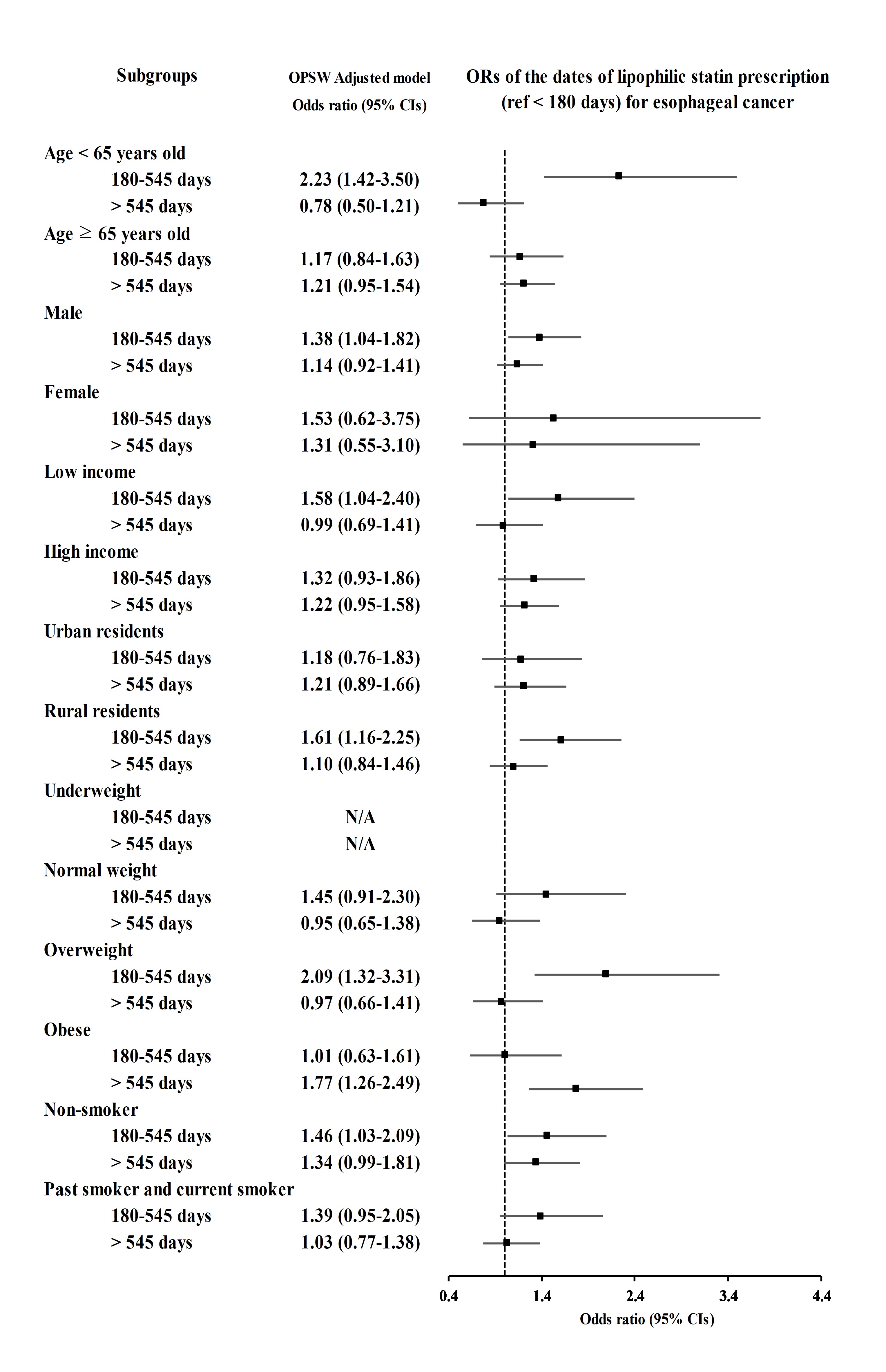

Supplement: Supplementary file 1 [file pharmaceuticals-16-00900-s001.zip › Fig S3A.JPG]

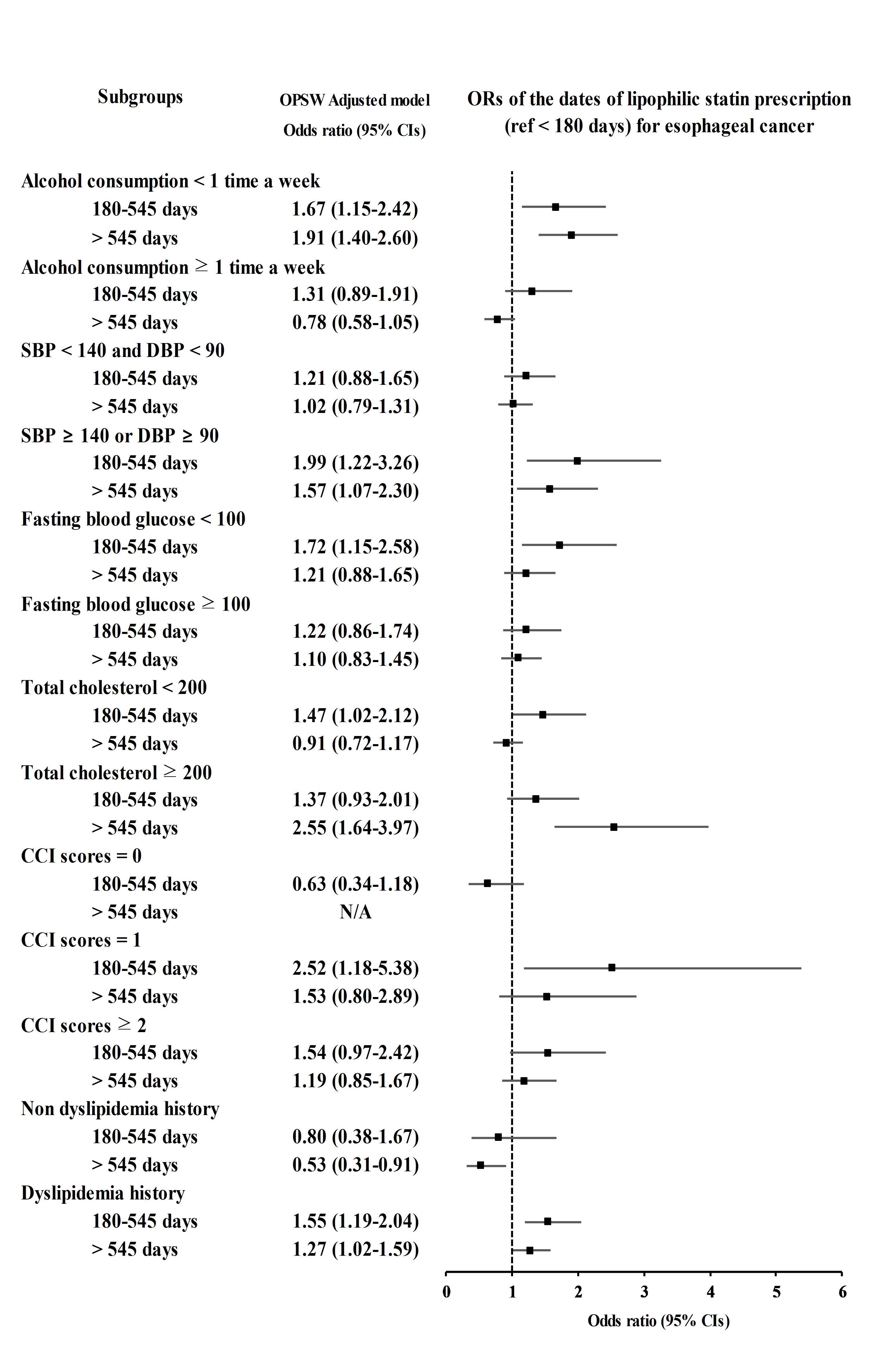

Supplement: Supplementary file 1 [file pharmaceuticals-16-00900-s001.zip › Fig S3B.JPG]

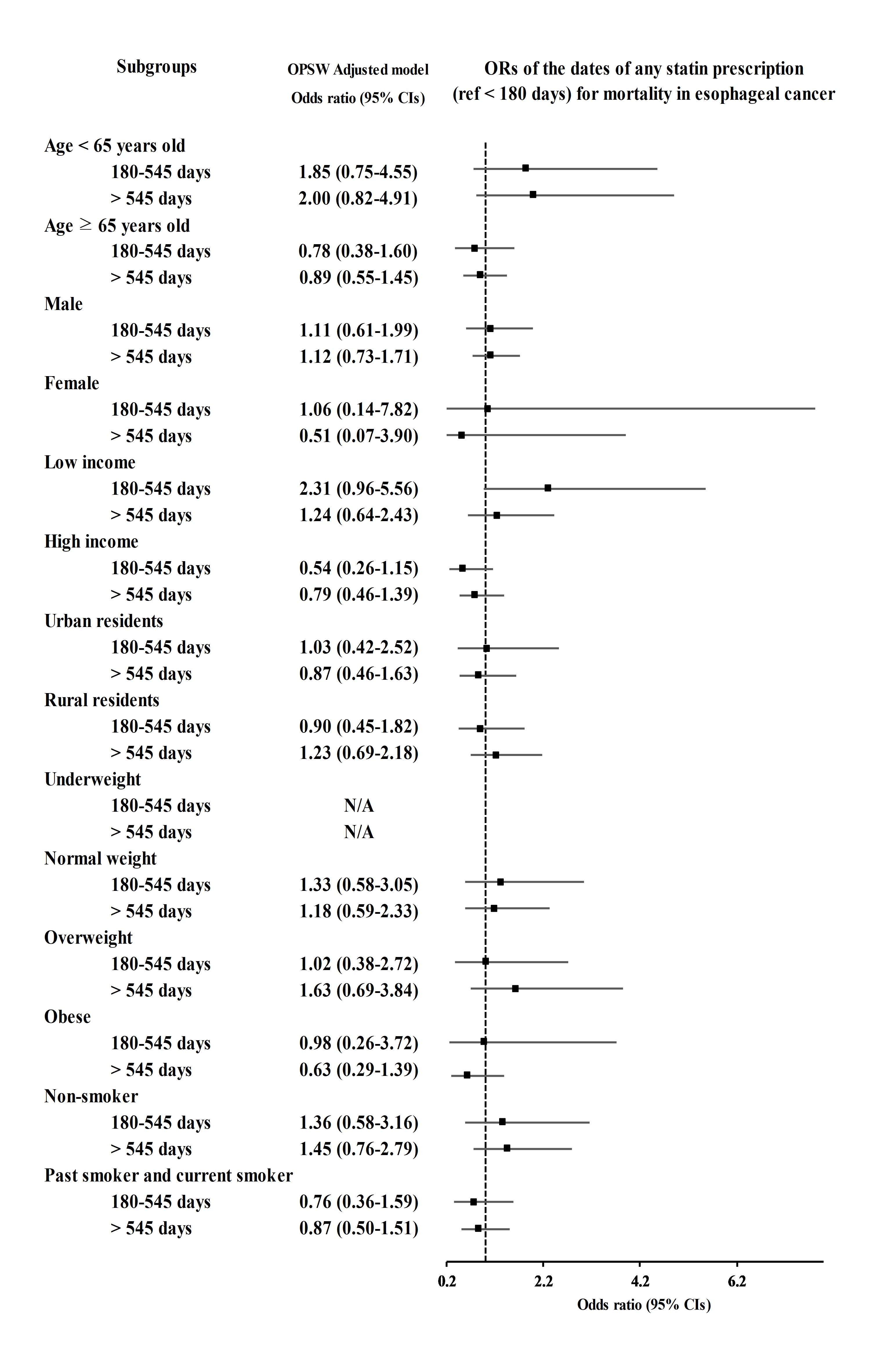

Supplement: Supplementary file 1 [file pharmaceuticals-16-00900-s001.zip › Fig S4A.JPG]

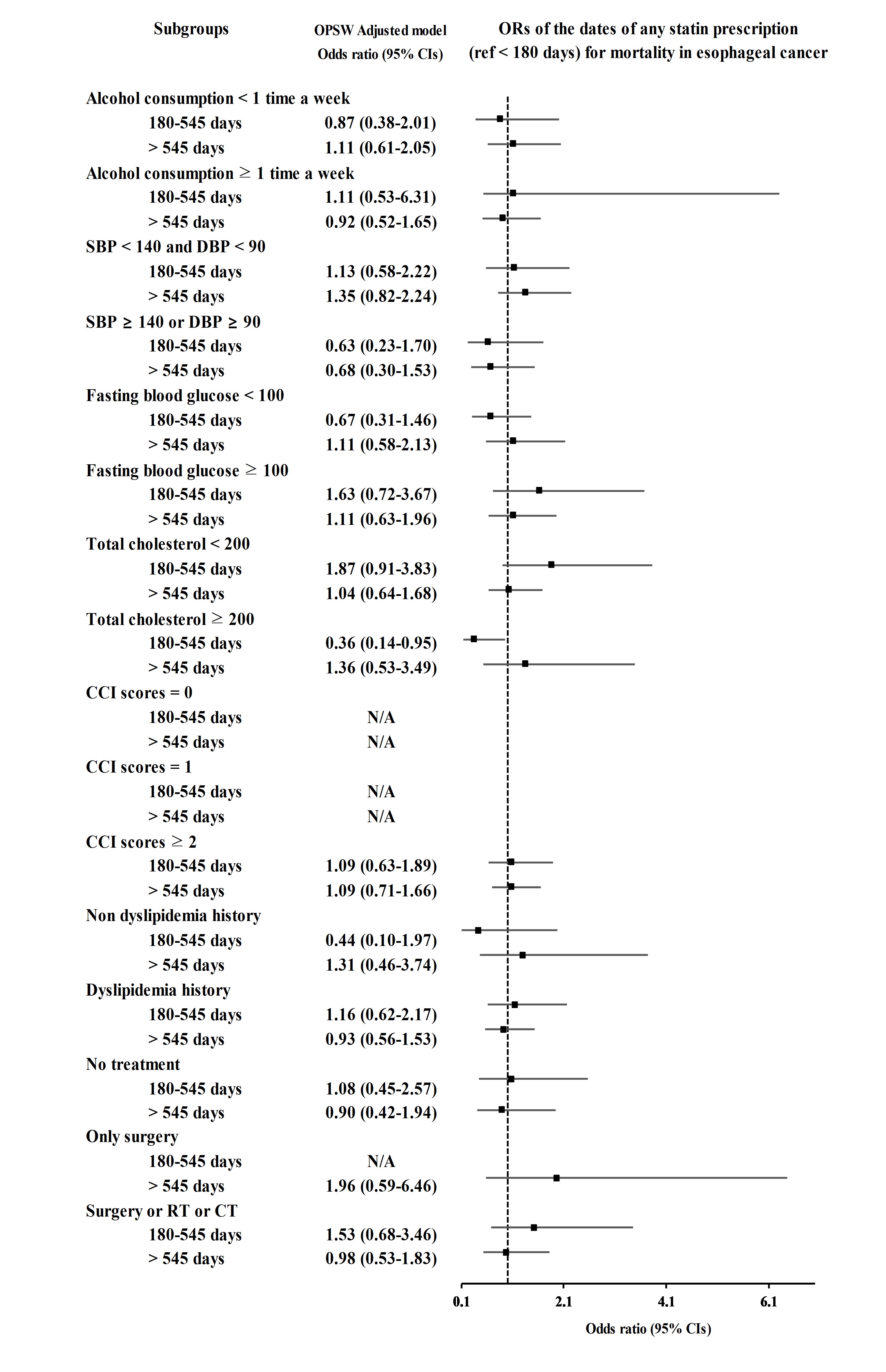

Supplement: Supplementary file 1 [file pharmaceuticals-16-00900-s001.zip › Fig S4B.JPG]

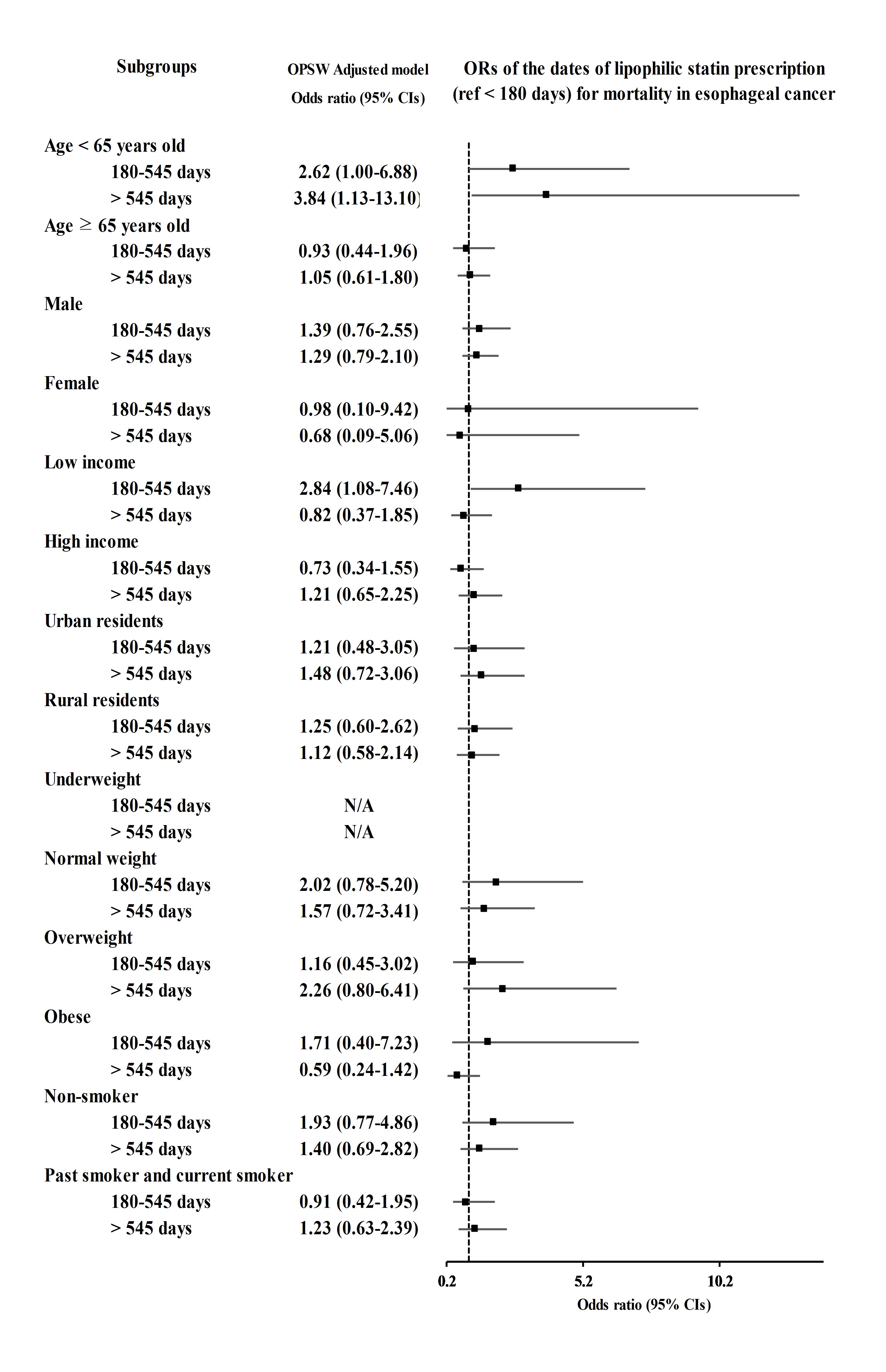

Supplement: Supplementary file 1 [file pharmaceuticals-16-00900-s001.zip › Fig S6A.JPG]

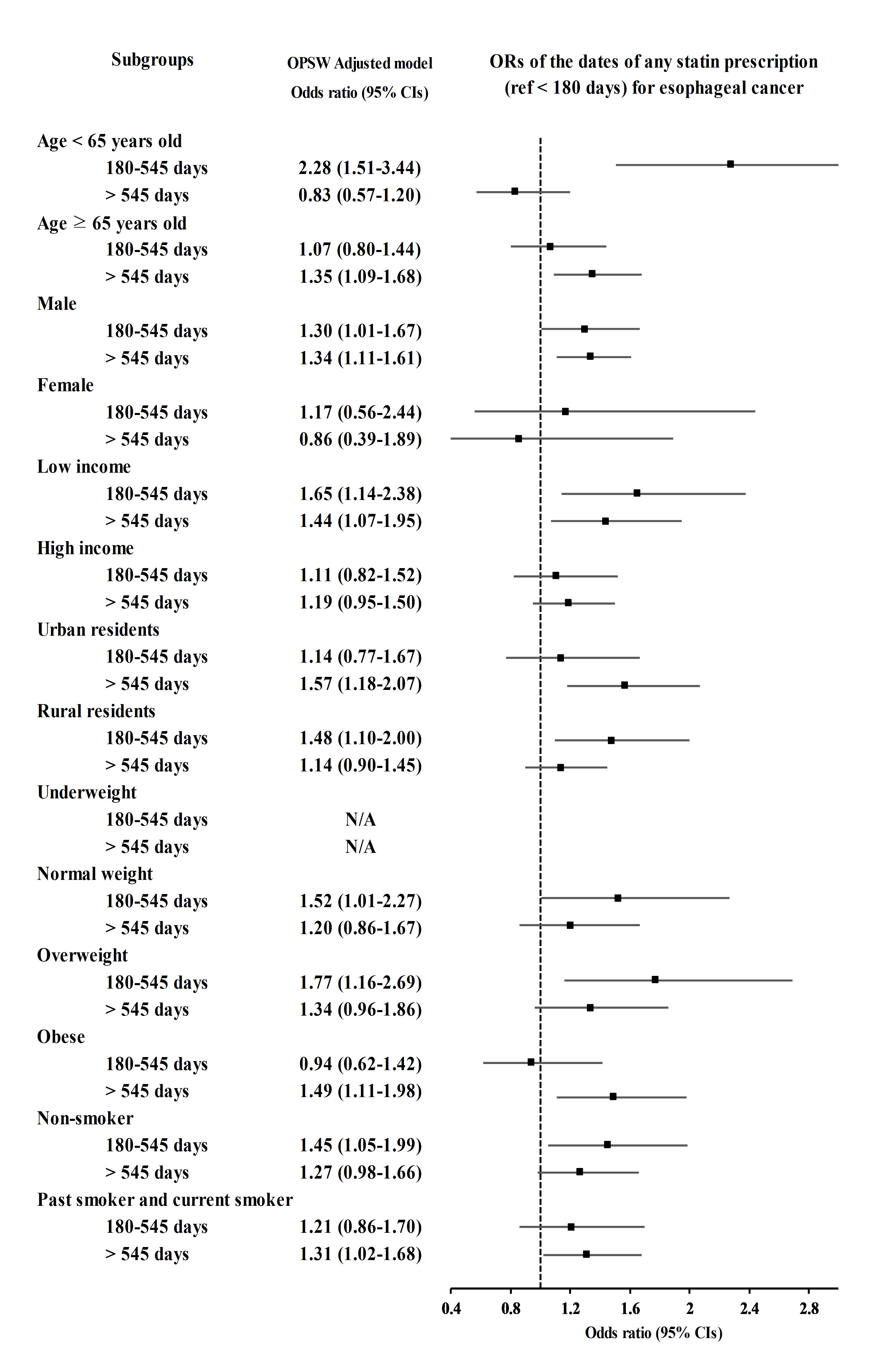

Supplement: Supplementary file 1 [file pharmaceuticals-16-00900-s001.zip › Fig. S1A.JPG]

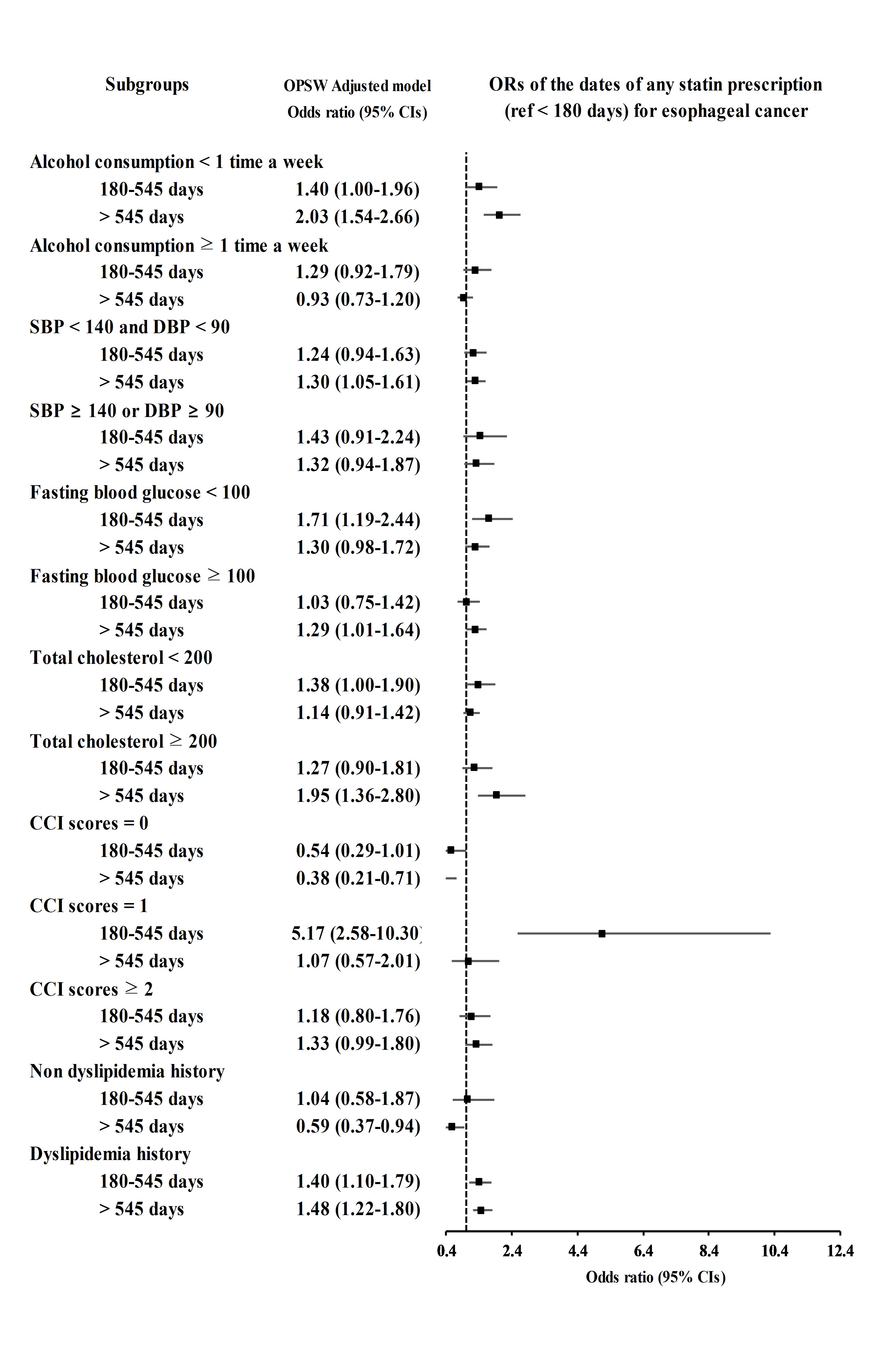

Supplement: Supplementary file 1 [file pharmaceuticals-16-00900-s001.zip › Fig. S1B.JPG]

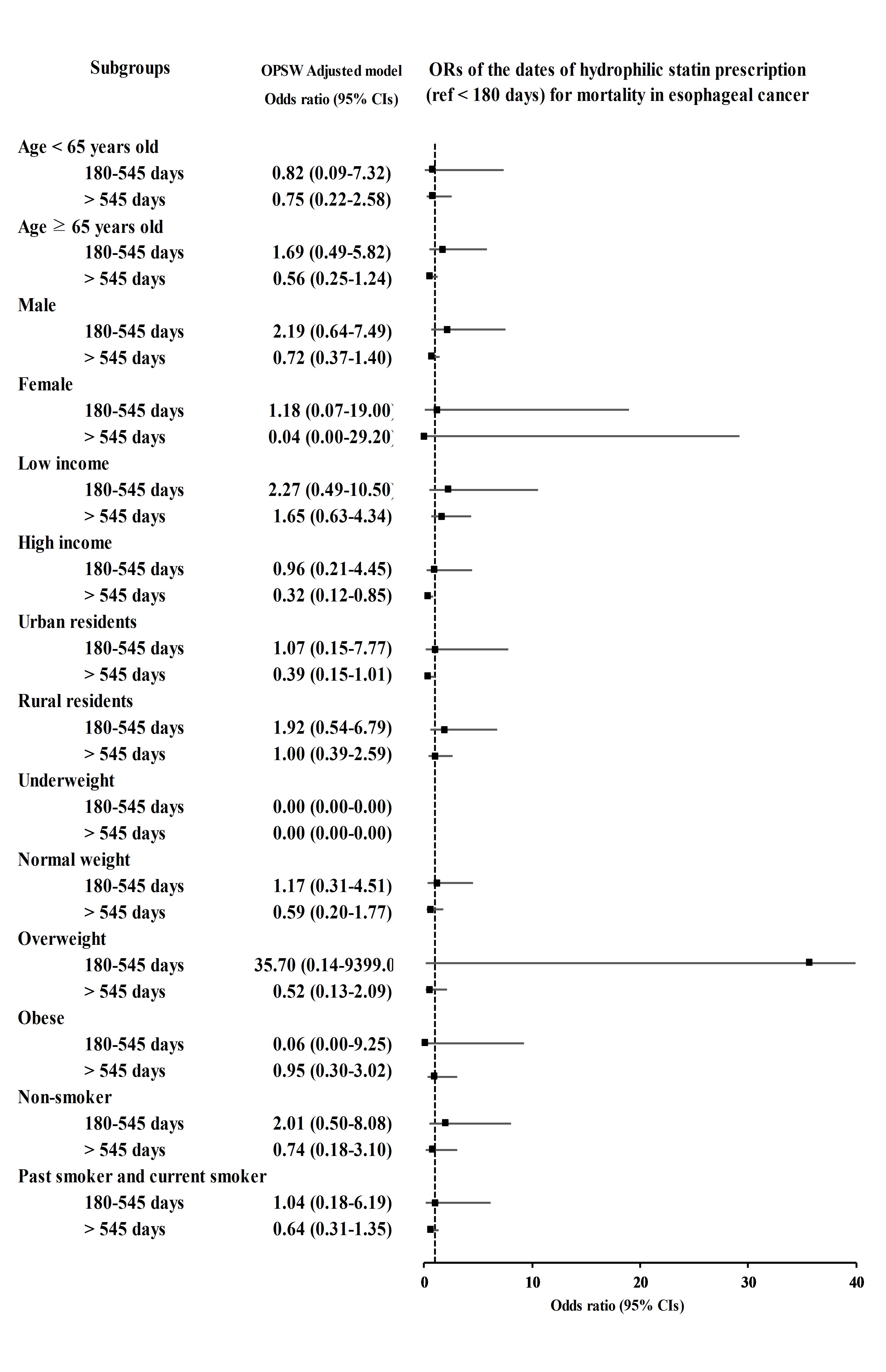

Supplement: Supplementary file 1 [file pharmaceuticals-16-00900-s001.zip › Fig. S5A.JPG]

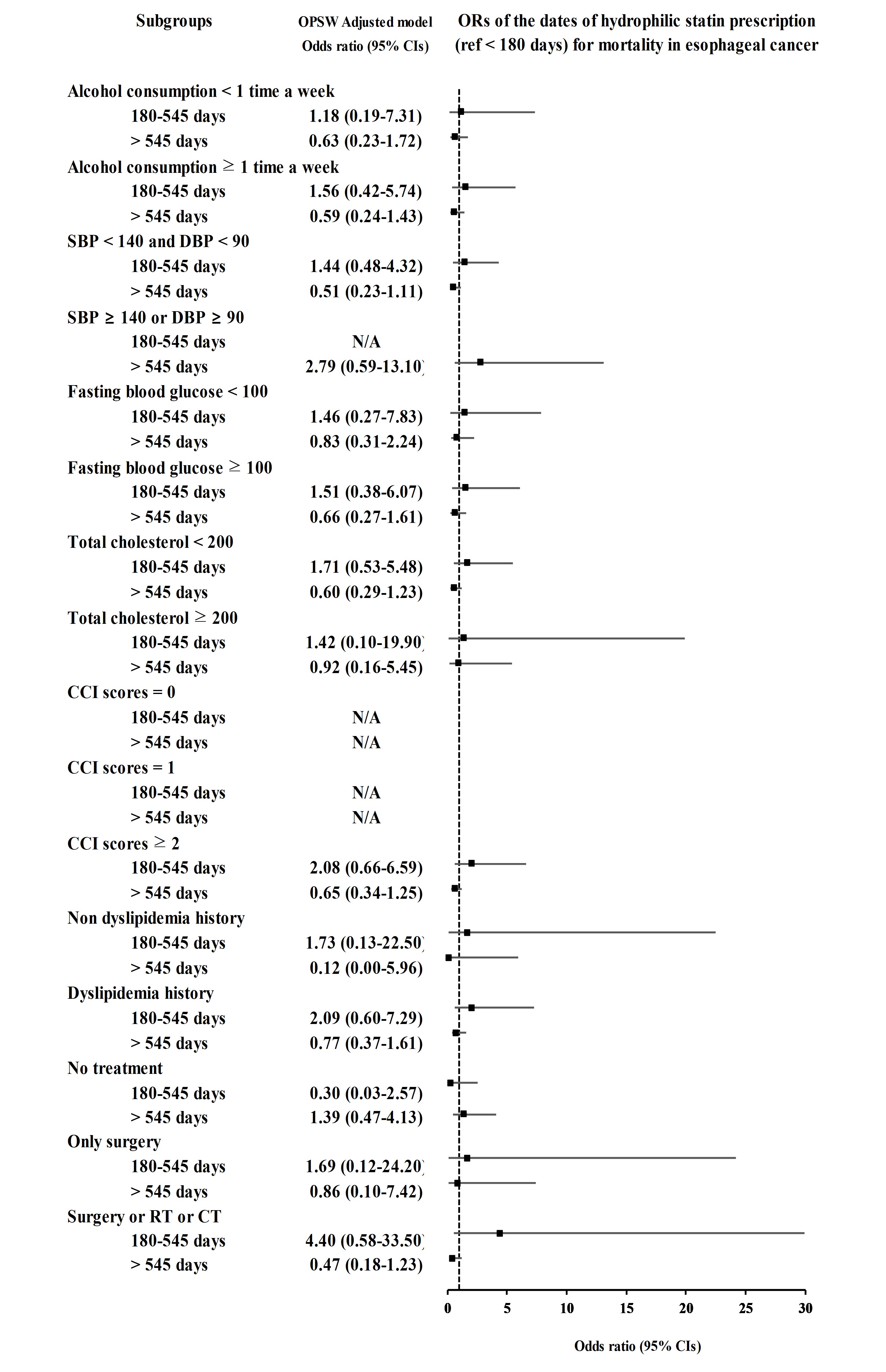

Supplement: Supplementary file 1 [file pharmaceuticals-16-00900-s001.zip › Fig. S5B.JPG]

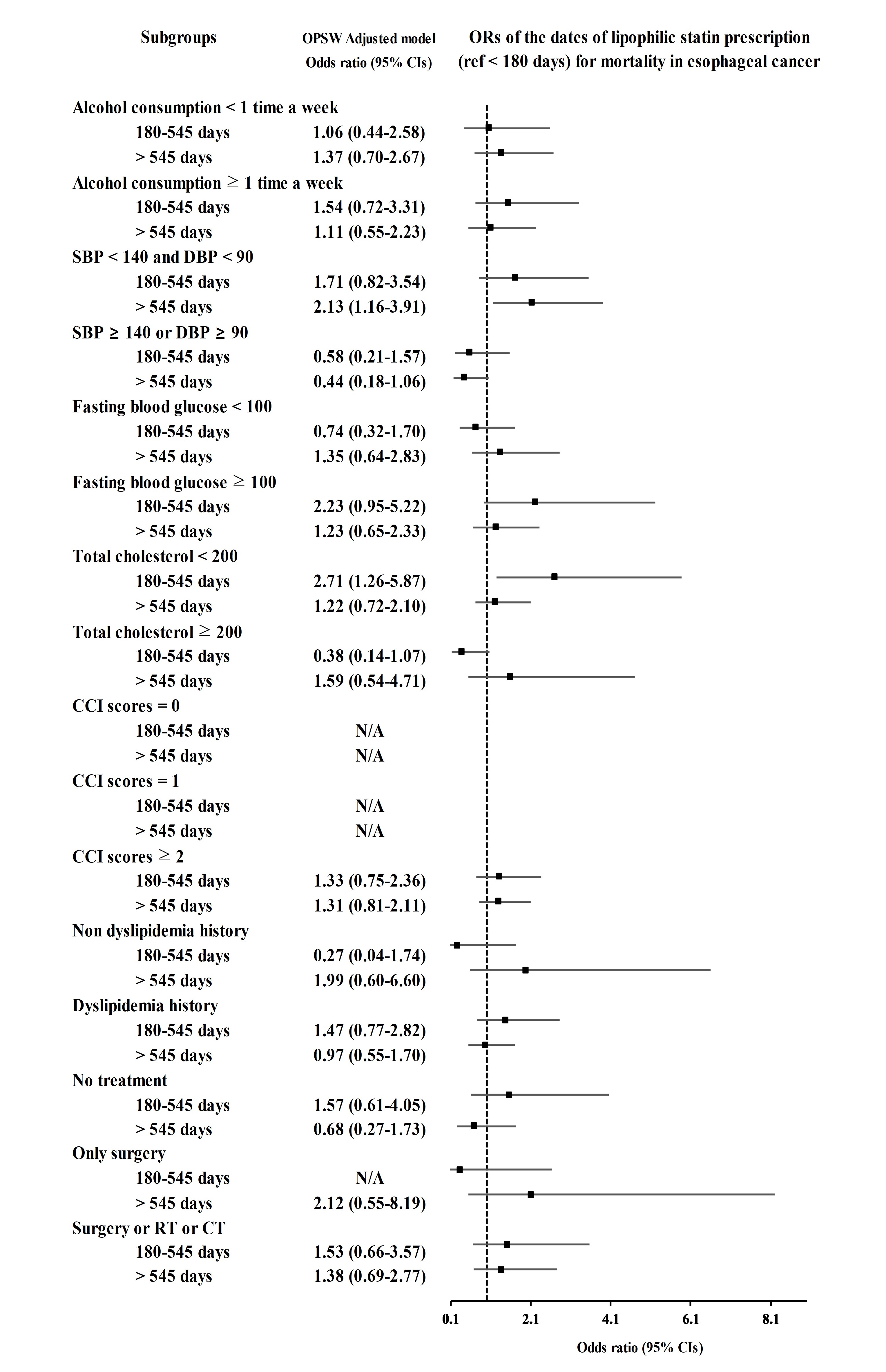

Supplement: Supplementary file 1 [file pharmaceuticals-16-00900-s001.zip › Fig. S6B.JPG]
